# Evaluating the restoration of Egypt’s Mediterranean Manzala Lagoon: a multi-index assessment of water quality and heavy metals

**DOI:** 10.1038/s41598-026-45115-x

**Published:** 2026-04-13

**Authors:** Manal A. Eissa, Seliem M. El Sayed, Fathy A. ElSayed, Ahmed Askalany, Mustafa Eissa, Mohamed E. Goher

**Affiliations:** 1 National Institute of Oceanography and Fisheries (NIOF), Cairo, 11694 Egypt; 2 Chemistry Department, Faculty of Science, Menoufia University, Shebin El-Kom, Egypt; 3 Faculty of Technology and Education, Sohag University, Sohag, Egypt; 4 Division of Water Resources and Arid Land, Hydrogeochemistry Department, Desert Research Center, Cairo, Egypt

**Keywords:** Manzala lake, Water quality assessment, Metal pollution indices, Eutrophication, Water quality, Principal component analysis, Ecology, Ecology, Environmental sciences, Hydrology

## Abstract

Lake Manzala, the largest coastal lagoon along Egypt’s Mediterranean delta, represents an ecosystem of exceptional ecological and socioeconomic significance, yet it has endured decades of severe environmental degradation. This study presents a comprehensive post‑restoration assessment of its water quality during 2021–2022 to evaluate the effectiveness of ongoing dredging and wastewater‑treatment initiatives. Twelve monitoring stations were systematically surveyed seasonally across the lake’s four sectors, and a wide array of physicochemical variables, nutrient salts, and heavy‑metal concentrations were determined. Integrated indices, including the Canadian Water Quality Index (CWQI), Aquatic Toxicity Index (ATI), Trophic State Index (TSI), Pollution Index (PI), and Heavy-Metal Pollution Index (HPI), were applied, alongside multivariate statistical analysis (Principal Component Analysis, PCA) to interpret spatial patterns and dominant controlling factors. Results revealed a persistent north–south gradient of deterioration, with the southern and southeastern sites receiving heavy loads of organic and inorganic pollutants from the Bahr El‑Baqar, Hadous, and Serw drains. The lake remains markedly hypereutrophic; mean BOD_5_ and COD values exceeded international thresholds, and high concentrations of Pb, Cd, Ni, and Cr indicate serious ecological risk. CWQI values (39–64) classified most areas as poor to marginal, while HPI values (> 180) indicate heavy‑metal pollution stress. PCA distinguished two principal drivers: **(1)** anthropogenic organic and metal loading, explaining about 52% of the total variance, and **(2)** natural oxygenation and self‑purification processes with a minor influence. Despite improved circulation from dredging and the commissioning of the Bahr El-Baqar wastewater treatment plant, overall chemical restoration remains incomplete. Long-term recovery requires catchment-wide nutrient control, sediment remediation, and continuous monitoring to restore the ecological balance and sustain the environmental and economic value of this vital wetland.

**Supplementary Information:**

The online version contains supplementary material available at 10.1038/s41598-026-45115-x.

## Introduction

Coastal lagoons are dynamic transitional interfaces between terrestrial freshwater and marine environments, characterized by complex hydrological and biogeochemical gradients^1^. These features render them extraordinarily productive yet highly sensitive to anthropogenic disturbances^2^. Unlike deep inland lakes, these shallow, wind-driven systems rely on variable salinity to regulate biodiversity and sediment–water interactions^3,4^. Functioning as the landscape’s natural kidneys, they filter and transform nutrients and contaminants before they reach the sea^5^. Globally, they provide vital ecosystem services, including carbon sequestration, shoreline stabilization, and critical habitats for valuable aquatic and avian species^6,7^. However, their proximity to dense human populations and industrial-agricultural hubs exposes them to severe anthropogenic pressures, driving widespread eutrophication, habitat loss, and ecological decline worldwide^8,9^.

Protecting these fragile ecosystems necessitates an integrated approach, as traditional single-parameter assessments fail to capture their complex spatial and temporal gradients^10,11^. Modern aquatic research addresses this by employing multi-index frameworks, such as the CWQI, ATI, TSI, PI, and HPI, to synthesize diverse physicochemical and biological data into standardized scores that quantify ecosystem health, trophic conditions, and pollution stress^12–17^. Furthermore, integrating multivariate statistical tools like PCA enables the clear differentiation of anthropogenic drivers from natural environmental variations^18,19^. Given the compounding effects of fluctuating marine exchange and climatic variability, continuous monitoring is vital. Tracking the accumulation of nutrients, organic matter, and heavy metals is critical to mitigate ecological degradation and human health risks associated with biomagnification in aquatic food webs^20,21^. Ultimately, this comprehensive assessment acts as both a diagnostic and preventive framework for the sustainable management of coastal resources.

Egypt’s northern Mediterranean coastline features five principal wetland lakes (Mariout, Edku, Burullus, Manzala, and the hypersaline Bardawil) that serve as vital ecological buffers against Nile Delta contaminants^22,23^. Historically contributing over 50% of Egypt’s fish production in the 1980s, their yield plummeted to approximately 11% by 2020 due to severe anthropogenic pollution, eutrophication, and habitat degradation, making them some of the most threatened aquatic systems in the Mediterranean^24,25^. This decline mirrors severe eutrophication, habitat loss, and the spread of invasive aquatic macrophytes^26^. Lake Manzala stands out as the largest and most socio‑economically important coastal brackish lake in Egypt. Geographically, it occupies the northeastern corner of the Nile Delta, bordered by the Mediterranean Sea to the north, the Suez Canal to the east, and cultivated delta lands to the south and west. The lake body, approximately 600 km² in area and averaging 2 m in depth, is separated from the sea by narrow sand barriers interrupted by natural inlets (boughazes), mainly El‑Gamil, New El‑Gamil, and El‑Qaboti^27,28^. Lake Manzala receives over 95% of its inflow (approximately 7,500 MCM annually) from six main drains: Bahr El-Baqar, Hadous, Serw, Ramsis, Faraskour, and Matariya. These mostly untreated discharges are heavily loaded with agricultural, industrial, and domestic pollutants^29,30^, driving the lake into a hypereutrophic state characterized by persistent algal blooms, severe hypoxia, and excessive sediment organic loading^25^. Concurrently, the lake has suffered a drastic morphological decline, shrinking from around 1,709 km^2^ in 1990 to about 572.4 km² in 2020, mainly due to land reclamation, illegal filling, and sedimentation^31^. Compounded by obstructed tidal inlets and local overfishing, these anthropogenic pressures have severely restricted hydrodynamic exchange, transforming this once highly productive coastal lagoon into one of Egypt’s most heavily polluted wetlands.

Recognizing the urgency of the situation, the Egyptian government launched the National Lake Restoration Program in 2017, prioritizing Lake Manzala for large-scale rehabilitation^31,32^. The strategy relies on two synergistic components to restore ecological and hydrological balance. The first component, hydrological restoration, involved extensive dredging campaigns to extract contaminated bottom sediments, deepen the basin, and reopen blocked inlets and silted channels^32^. These interventions were also accompanied by the removal of dense aquatic macrophytes that had overgrown many sections of the lake, thereby enhancing water circulation, increasing oxygen exchange, and reducing the internal recycling of nutrients within the sediment–water interface^25^. Evaluating restoration success in this complex transitional ecosystem requires moving beyond isolated chemical tests to an integrated multi-index framework. Combining comprehensive indices, namely, the Canadian Water Quality Index (CWQI), Aquatic Toxicity Index (ATI), Trophic State Index (TSI), Pollution Index (PI), and Heavy-Metal Pollution Index (HPI), with multivariate tools like Principal Component Analysis (PCA) provides a holistic assessment of the lake’s physicochemical health, trophic state, and heavy-metal contamination^33,34^ This robust methodology effectively pinpoints pollution ‘hot-spots,’ tracks spatio-temporal variations, and distinguishes anthropogenic impacts from natural environmental drivers.

The main objectives of this study extend beyond a localized monitoring effort to diagnose the recovery trajectories and environmental elasticity of Lake Manzala during the 2021–2022 post-restoration phase. Specifically, the research conducts a comprehensive evaluation of the spatial and seasonal variability of key physicochemical parameters, nutrients, and heavy metals to determine the lake’s overall status. By integrating multiple indices (CWQI, ATI, TSI, PI, and HPI), the study seeks to unravel the complex interplay between legacy pollution and modern anthropogenic inflows, assessing the lake’s suitability for sustaining aquatic life. Furthermore, through multivariate statistical analyses, particularly PCA, the study interprets the relationships among water quality parameters to identify the dominant factors influencing pollution patterns and localized hotspots. Ultimately, this work evaluates the environmental response to large-scale dredging and wastewater treatment interventions, providing transferable scientific insights into the lag time between physical intervention and geochemical recovery. By achieving these objectives, this research not only contributes to a deeper understanding of the post-restoration dynamics of Egypt’s largest coastal lagoon but also offers a scientifically grounded blueprint for the integrated monitoring and sustainable management of similar anthropogenically impacted brackish ecosystems across the Mediterranean Basin and worldwide.

## Materials and methods

### Study area

Lake Manzala is the largest coastal wetland in the Nile Delta, located in the northeastern Nile Delta of Egypt, situated between latitudes 31°07′03.2ʺ N – 31°23′53.7ʺ N and longitudes 31°47′45.4ʺ E – 32°14′35.0ʺ E^32^. It is bounded by the Mediterranean Sea to the north, the Suez Canal to the east, the Damietta Branch of the Nile to the west, and the extensive agricultural lands of Dakahlia and Sharqia governorates to the south.

Historically, the lake covered an area of approximately 1,709 km^2^ in 1900 but underwent severe shrinkage due to extensive land reclamation and drying processes, reaching a minimum of 565.9 km^2^ in 2016. However, following the comprehensive national restoration and dredging program initiated in 2017, the lake’s total area marginally increased to approximately 572.4 km^2^ in 2020, with open water constituting about 75% of this area^29^. The lake is a rectangular, shallow, turbid, and brackish water body.

Hydrologically, the lake functions as a semi-enclosed system maintaining connectivity with the Mediterranean Sea through three primary tidal inlets (Boughazes): Al-Gamil, Ashtoum Al-Gamil, and Al-Sofara, in addition to the El-Qabouti Canal, which links the lake to the Suez Canal^28^. The Al-Sofara inlet, situated in the northwestern corner, plays a critical role in hydrodynamic exchange by facilitating the influx of seawater into the lake basin via several channels, most notably the El-Baghdady opening in the north-central sector. These connections facilitate the exchange of water and biota between the lake and the sea. These inlets play a crucial role in regulating water circulation, salinity, and ecological balance within the lake and are considered the principal pathways for water renewal between Lake Manzala and the Mediterranean, and they significantly contribute to improving the lake’s water quality through enhanced flushing and seawater exchange^27^. Conversely, the southern and western sectors receive massive inflows of drainage water (estimated at > 7000 MCM year^1^), mixed with sewage and industrial effluents via major drains, principally Bahr El-Baqar, Hadous, Al-Serw, and Ramsis. Additionally, the lake is hydraulically linked to the Damietta Branch of the Nile River via the El-Inaniya Canal, allowing the southwestern sector of the lake to receive freshwater input from the Serw and Faraskour pumping stations. This dual influence of marine inlets in the north and nutrient-rich drainage in the south creates distinct ecological zones within the lake.

**Table 1:** Details and coordinates of the sampling locations in El-Manzala Lake.

Sector	Stations	Depth (m)	Latitude *N*	Longitude E
North	1	5.2	31^°^ 16^’^ 7.53^”^	32^°^ 12^’^ 39.20^”^
	2	1.5	31^°^ 14^’^ 49.66^”^	32^°^ 11^’^ 58.78^”^
	3	5.0	31^°^ 17’ 17.97^”^	32^°^ 09^’^ 53.49^”^
	4	4.5	31^°^ 21^’^ 2.59^”^	32^°^ 00^’^ 22.73^”^
Middle	5	1.3	31^°^ 16^’^ 4.88^”^	32^°^ 03^’^ 42.61^”^
	6	1.5	31^°^ 16^’^ 31.33^”^	32^°^ 00^’^ 49.55^”^
East	7	1.8	31^°^ 12^’^ 2.64^”^	32^°^ 12^’^ 11.01^”^
South	8	1.5	31^°^ 11^’^ 5.18^”^	32^°^ 04^’^ 54.62^”^
	9	1.3	31^°^ 10^’^ 24.25^”^	32^°^ 04^’^ 28.71^”^
	10	1.3	31^°^ 11^’^ 25.20^”^	32^°^ 02^’^ 21.13^”^
West	11	1.2	31^°^ 15^’^ 14.21^”^	31^°^ 51^’^ 29.45^”^
Western north	12	1.3	31^°^ 22^’^ 11.01^”^	31^°^ 53^’^ 17.53^”^

**Fig. 1:**
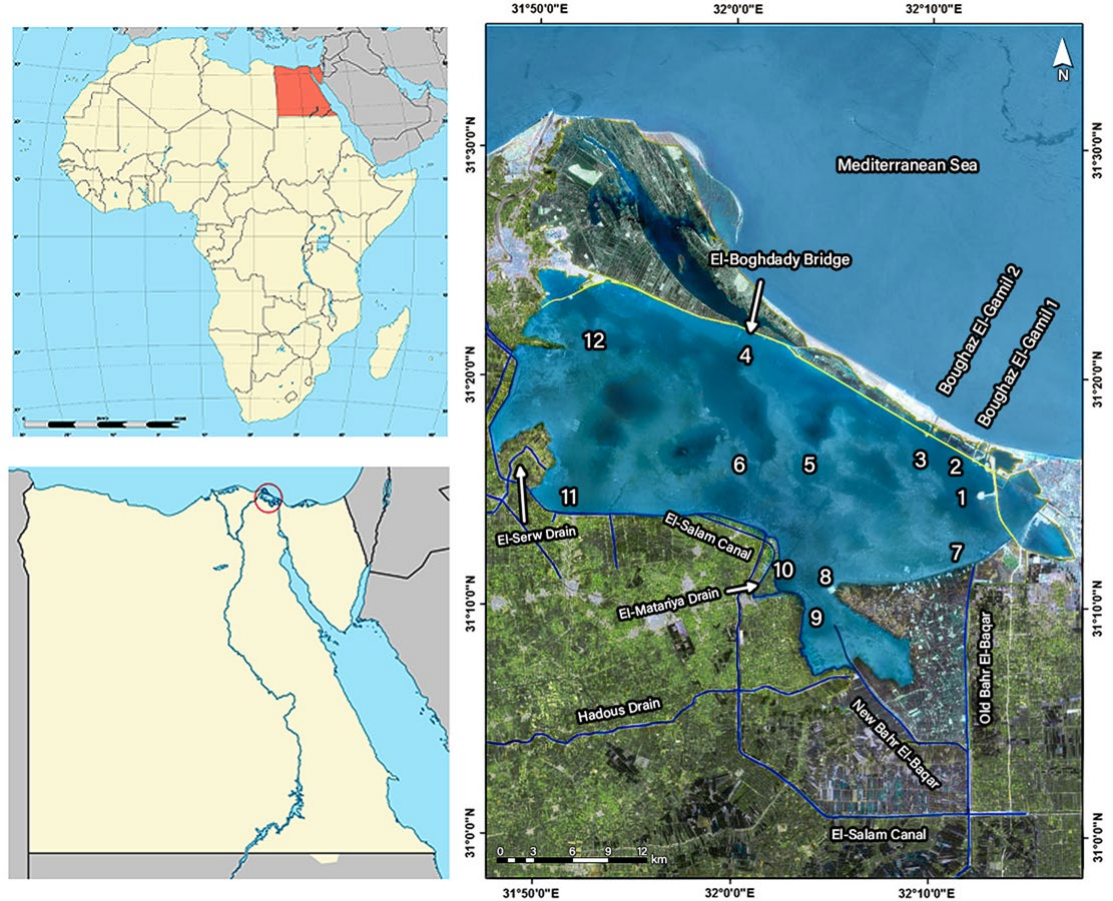
Map of Lake Manzala showing the geographical location and distribution of sampling sites, modified after^29^ (The map was generated using ArcGIS Pro 3.1.0, https://www.esri.com/en-us/arcgis/products/arcgis-pro).

### Water sampling and analysis

Four seasonal sampling campaigns were conducted during 2021–2022 across 12 strategic locations representing Lake Manzala’s hydrological and pollution gradients (Table 1; Fig. 1). At each site, in situ parameters including temperature, electrical conductivity (EC), dissolved oxygen (DO) and pH were measured using a calibrated Hydrolab Multiparameter Probe (Multiset 430i, WTW, Germany) to ensure data accuracy^35^. Water transparency was determined using a standard 30-cm diameter Secchi disk. Subsurface water samples were collected using a 2 L Ruttner sampler at a depth of approximately 0.5 m. The collected aliquots were immediately transferred into pre-cleaned polyethylene bottles, which were stored at 4 °C in ice boxes to maintain sample integrity during transport. Upon arrival at the laboratory, samples were processed immediately after filtration through 0.45 μm membrane filters. Samples for biochemical oxygen demand (BOD_5_) were collected in glass-stoppered bottles to prevent atmospheric interference. For heavy metal analysis, water subsamples were collected in acid-washed bottles and preserved by acidification with ultrapure nitric acid (HNO_3_) to pH < 2 to prevent metal adsorption to container walls.

#### Laboratory analyses and quality control

Chemical oxygen demand (COD) was determined via potassium permanganate oxidation. Salinity was quantified gravimetrically (as Total Dissolved Solids) by evaporating filtered samples and drying the residue at 180 °C until constant weight. Nutrient concentrations (ammonia, nitrite, nitrate, total nitrogen, orthophosphate, and total phosphorus) were measured using a double-beam UV-visible spectrophotometer (Jenway 680, UK) at specific wavelengths following standard colorimetric methods^35,36^. For Chlorophyll-a (Chl-a), samples were filtered (0.7 μm GF/F), extracted in 90% acetone as per^37^, and calculated spectrophotometrically per^38^. Heavy metals were analyzed using an atomic absorption spectrophotometer (SavantAA AAS with GF-5000 Graphite Furnace) according to standard protocols^32^. To guarantee the reproducibility and analytical precision of all physicochemical, nutrient, and heavy-metal measurements, triplicate analyses were performed for every sampling location, and the results were expressed as mean values. A summary of the experimental procedures and instrumentation is provided in Table 2.

**Table 2:** Summary of the experimental procedures and instruments used in the chemical analyses.

Parameter	Method	Instrument/model	References
Temp., EC, pH, DO	Multiparameter probe, in situ measurement	Hydrolab Multiset 430i, WTW, Germany	^35^
Salinity, TSS	Gravimetric evaporation	Laboratory oven + analytical balance	^35^
BOD_5_	5-day incubation at 20 °C	Glass-stoppered BOD bottles	^35^
COD	Potassium permanganate oxidation	Laboratory chemical digestion setup	^35^
Nitrite and nitrate	Colorimetric measurement at 543 nm	Jenway UV–Vis 680, UK	^35^
Ammonia	Phenate method at 640 nm	Jenway UV–Vis 680, UK	^35^
Orthophosphate	Ascorbic acid–molybdate method at 880 nm	Jenway UV–Vis 680, UK	^35^
Total nitrogen (TN)	Persulfate digestion + nitrate determination	Digestion block + UV–Vis	^36^
Total phosphorus (TP)	Persulfate digestion + phosphate determination	Digestion block + UV–Vis	^36^
Chlorophyll-a	Filtrated and extracted in 90% acetone	Jenway UV–Vis 680, UK	^37,38^
Heavy metals	Atomic absorption spectrophotometry	SavantAA AAS + GF-5000 Graphite Furnace	^35^

### Water quality and pollution indices

#### Water quality indices

Water quality indices (WQIs) provide an integrated and simplified representation of the overall status of a water body by converting a large set of physicochemical and biological data into a single, interpretable score. This approach enables scientists, environmental managers, and decision-makers to evaluate water conditions efficiently and to compare spatial and temporal variations in water quality^12–14^. The concept of the WQI is especially useful in highly complex aquatic systems such as lakes, rivers, and groundwater aquifers, where numerous interacting parameters determine ecological health. The general principle behind the WQI is that individual water quality parameters, such as nutrients, dissolved oxygen, pH, turbidity, organic load, and toxic contaminants, are normalized against standard permissible limits and then mathematically aggregated into a composite index. When the values of any of these parameters exceed acceptable thresholds, the overall WQI score declines, signaling possible ecological degradation or risk to human health^33^. Thus, the WQI serves not only as a diagnostic tool but also as a monitoring framework that helps in environmental planning, pollution control, and assessing suitability for specific uses such as drinking, irrigation, fisheries, or recreation^39^.

In the present study, the water quality of Lake Manzala was assessed using three complementary indices to capture different ecological and chemical dimensions of water quality: (1) the Canadian Water Quality Index (CWQI), (2) the Aquatic Toxicity Index (ATI), and (3) the Trophic State Index (TSI). Together, these three indices offer a comprehensive and multidimensional assessment of the ecological condition of Lake Manzala, capturing aspects of general water quality, chemical toxicity, and nutrient-driven productivity. Their combined application enhances the reliability and interpretability of the water quality evaluation and provides a strong scientific basis for environmental management and restoration planning. The specific parameters integrated into each water quality index, along with their respective environmental diagnostic purposes, are summarized in Table 3.

**Table 3:** Summary of parameters used for the environmental indices used.

Index	Parameters Included	Environmental Significance
CWQI	Temp, TSS, pH, DO, NH_4_^+^, NO_2_^−^, NO_3_^−^, and HMs.	General water suitability for aquatic life.
TSI	Chl-a, TP, TN, and Secchi Depth (SD).	Trophic status and biological productivity.
ATI	pH, DO, PO_4_^3−^, NH_4_^+^, Cu, Mn, Ni, Pb, Zn,	Potential toxic effects on aquatic organisms
HPI / PI	Studied HMs (Cd, Cu, Cr, Fe, Mn, Ni, Pb, Zn).	Heavy metal contamination and pollution intensity.

##### Canadian water quality index

The Canadian Water Quality Index (CWQI) was utilized to synthesize complex physicochemical and metallic data into a single score (0–100) (Table 4)^40^. The index calculation followed the CCME standard protocols, with detailed equations and normalization procedures provided in the supplementary material (section S1).

**Table 4:** CCME water quality index (WQI) categories and their descriptive ratings according to CCME user’s manual^40^.

CCME-WQI value	Rating	Description
0–44	Poor	Water quality is almost always threatened or impaired; conditions typically deviate substantially from natural or desirable levels.
45–64	Marginal	Water quality is frequently threatened or impaired; conditions often depart from natural or desirable levels.
65–79	Fair	Water quality is generally protected, although occasionally threatened or impaired; conditions sometimes deviate from natural or desirable levels.
80–94	Good	Water quality is protected with only minor threats or impairments; conditions rarely deviate from natural or desirable levels.
95–100	Excellent	Water quality is protected with an almost complete absence of threat or impairment; conditions remain very close to natural or pristine levels.

##### Aquatic toxicity index (ATI)

The Aquatic Toxicity Index (ATI) was applied to evaluate the potential toxic effects of contaminants on the lake’s ecological integrity by^41^. The index was computed using a modified unweighted additive aggregation function to integrate the biological responses of resident organisms into a single quantitative value, with scores ranging from 0 (higher potential toxicity) to 100 (negligible toxic effects) Table 5^42^. Detailed calculation procedures and parameter quality ratings $\:\left({q}_{i}\right)\:$are described in the supplementary material (section S2).

**Table 5:** Aquatic toxicity index (ATI) classification and corresponding water quality ratings according to^41^.

ATI range	Water quality rating
0–50	Completely unsuitable for sustaining normal fish life
51–59	Suitable only for hardy and highly tolerant fish species
60–100	Suitable for supporting all major fish species and normal aquatic life

##### Trophic state index (TSI)

The Trophic State Index (TSI) was applied to evaluate nutrient enrichment and biological productivity in Lake Manzala, following the integrated framework of^43,44^. The index was calculated based on the annual mean values of four key variables: Secchi depth (SD), chlorophyll-a (Chl-a), total phosphorus (TP), and total nitrogen (TN)^45^. This multi-parameter approach utilizes logarithmic relationships to determine the lake’s trophic status on a scale from 0 to 100 (Table 6) as detailed in the supplementary material (section S3, Table S1).

**Table 6:** TSI classification and ecological description according to^43,46^.

TSI range	Trophic category	Ecological description
0–30	Ultra-oligotrophic	Extremely clear water, very low nutrient concentrations, minimal algal biomass, high transparency; typical of unpolluted, pristine lakes.
31–40	Oligotrophic	Low productivity, clear water, limited nutrient availability, low chlorophyll-a, generally high dissolved oxygen throughout the water column.
41–50	Mesotrophic	Moderate nutrient levels, moderate algal biomass, balanced productivity; transitional phase, may shift toward eutrophic under increased nutrient loading.
51–60	Lower Eutrophic	Increasing productivity, noticeable algal growth, moderate decline in water transparency; early signs of eutrophication.
61–70	Eutrophic	High nutrient enrichment, frequent algal blooms, marked reduction in Secchi depth, increased risk of oxygen depletion in bottom waters.
71–80	Hypereutrophic	Heavy algal blooms possible throughout summer; dense macrophyte beds; hypereutrophic
> 80	Severe Hypereutrophic	Algal scums; summer fish kills; few macrophytes due to algal shading; rough fish dominance

#### Metal pollution indices

Heavy metals are among the most persistent and hazardous pollutants in aquatic environments due to their toxicity, non-biodegradability, and ability to bioaccumulate through the food chain. Because their ecological and health impacts depend not only on their absolute concentrations but also on their combined effects, several heavy metal pollution indices have been developed to provide an integrated assessment. These indices condense complex datasets into a single numerical value, enabling a clearer interpretation of contamination levels, identification of pollution sources, and improved decision-making for water quality management.

##### Pollution index (PI)

The Pollution Index (PI) was employed to assess the intensity of heavy metal contamination by comparing measured concentrations (Ci) against their respective international water quality standards (Si)^47,48^. This index provides a standardized score to classify pollution levels into five categories, ranging from low to severe^49^ (Table 7). The specific calculation formula and the safety thresholds used for each metal are provided in the supplementary material (section S4).

**Table 7:** Classification of the pollution index (PI) for heavy metals according to^49^.

PI range	Pollution category	Description
PI < 1	Low pollution	Metal concentrations are within permissible limits; no significant contamination is detected.
1–2	Medium pollution	Slight elevation above standards; water quality shows early signs of deterioration.
2–3	High pollution	Noticeable contamination: heavy metals exceed permissible levels and may begin affecting aquatic life.
3–5	Very high pollution	Strong metal contamination: water is unsuitable for most ecological functions.
> 5	Severe pollution	Heavy metal levels pose serious risk to aquatic ecosystems and human use.

##### Heavy metal pollution index (HPI)

The Heavy Metal Pollution Index (HPI) was used to determine the cumulative toxic burden of trace elements in Lake Manzala^50^ (Table 8). This index integrates monitored concentrations (Mi) with their corresponding ideal (Ii) and standard (Si) values using a weighted arithmetic mean method^51^. A critical threshold of 100 was applied to distinguish between suitable and unsuitable water quality, with full details of the sub-index (Qi) and unit weight (Wi) calculations provided in the supplementary material (section S5).

**Table 8:** Heavy metal pollution index (HPI) rating scale.

HPI value	Pollution status	Interpretation
< 50	Low heavy metal pollution	Water quality is generally safe; metals are well below critical limits.
50–100	Moderate pollution	Rising contamination levels: careful monitoring is required.
≥ 100	High/critical pollution	Water is unsuitable for aquatic life due to excessive heavy metal load.

### Principal component analysis (PCA)

Principal Component Analysis (PCA) was performed to identify the underlying structure governing the variability in the dataset and to detect potential pollution sources variance^52–56^. Prior to analysis, the data were standardized (Z-score normalization), and the principal components (PCs) were extracted based on the Kaiser criterion (eigenvalues > 1) using OriginPro 2018 software. This approach enabled the interpretation of spatial and temporal patterns through loading matrices and biplots, accounting for the maximum cumulative variance explained. The details about PCA are provided in the supplementary material (section S6). The PCA data matrix was constructed using the full raw dataset, encompassing all seasonal measurements across the 12 sampling stations. This global PCA approach was employed to provide a comprehensive overview of the underlying structure governing water quality variability throughout the study period, without averaging the data.

### Statistical analysis

All statistical analyses were performed using XLSTAT software (version 2016.02.28451). Prior to testing, data normality and homogeneity of variance were verified using Shapiro–Wilk and Levene tests, respectively; non-normal data were log-transformed to satisfy parametric assumptions. A one-way Analysis of Variance (ANOVA) was applied to evaluate spatial and temporal differences, followed by Tukey’s HSD post hoc test for pairwise comparisons (*p* < 0.05 and *p* < 0.01). Additionally, Pearson’s correlation analysis was conducted to examine relationships among variables and identify potential common pollution sources^57,58^. Parameters showing a correlation coefficient higher than 0.90 were evaluated for redundancy. Consequently, EC and COD were omitted from the PCA model in favor of salinity and BOD_5_ to prevent the artificial inflation of variance associated with ionic parameters.

## Results and discussion

### Physical and chemical parameters

The physicochemical characteristics of Lake Manzala exhibited pronounced spatial and temporal variations, reflecting the combined effects of drainage inflows, marine water exchange, and ongoing hydrological restoration. Comprehensive datasets detailing the physicochemical characteristics, nutrient concentrations, organic loads, and heavy metals across all sampling stations and seasons are provided in the Supplementary Material (Tables S1–S5**)**. These tables include seasonal ranges and annual means (± SD) for each sampling station that support the spatial and temporal analyses discussed in this study. A general descriptive summary of these parameters is further presented in Table 9. Water temperature in Lake Manzala exhibited a typical seasonal pattern, primarily controlled by the Mediterranean climatic conditions and the lake’s shallow bathymetry. This shallowness facilitates rapid thermal equilibrium with the atmosphere, a phenomenon also observed in other Northern Deltaic lakes like Burullus and Edku^46,59^. Significantly, the elevated summer temperatures (exceeding 30 °C) (Table 9) serve as a catalyst for accelerated biochemical kinetics, including microbial respiration and nitrification. This thermal stress, combined with organic loading, exacerbates dissolved oxygen depletion, and influencs nutrient regeneration in eutrophic water bodies during warmer months^60^.

The lake showed a pronounced spatial gradient in transparency, with visibility severely restricted in the southern sectors compared to the northern zones. This persistent turbidity is not merely a function of depth but reflects a complex interplay between high nutrient-driven phytoplankton blooms and the mechanical resuspension of fine sediments by wind—a common feature in shallow Mediterranean lagoons^32,46,59^. The low Secchi disk readings in the south (averaging < 10 cm) underscore the impact of heavy drainage inflows, which restrict light penetration, thereby limiting the photic zone and hindering the recovery of submerged macrophytes^46^. These findings are further supported by the high concentrations of total suspended solids (TSS), which reached their highest value in the southern stations (e.g., ST7 and ST9). A significant negative correlation (*r* = -0.61) was observed between transparency and TSS, confirming that the elevated suspended load originates from the drainage discharge.

A strong positive correlation was established between EC and salinity (*r* = 0.98, *p* < 0.01), identifying salinity as the governing factor of the lake’s ionic strength^61^. To ensure statistical robustness and avoid redundancy in multivariate models, salinity was prioritized for further analysis. The spatial distribution revealed a clear seawater influence (boughazes) in the north, contrasted by freshwater dominance from agricultural drains in the southwest. This fluctuating salinity regime classifies Lake Manzala as a dynamic transitional system, where the balance between marine intrusion and drainage discharge dictates the osmotic stress on aquatic biota sources^62,63^.

**Table 9:** Descriptive statistics of physicochemical parameters in Lake Manzala (2021–2022).

Parameters	Range	Mean ± SD	Permissible level
Temp. °C	12.61 – 31.46	22.65 ± 0.35	8-28^a^
Trans cm	5 – 75	26.71 ± 6.32	
TSS mg/L	56.46 – 126.39	62.27 ± 21.22	25^a^
EC mS/cm	2.11 – 54.34	14.38 ± 6.99	
Salinity psu	0.89 – 38.73	10.05 ± 4.64	
pH	7.4 – 9.28	8.5 ± 0.139	6.5-9^a^
DO mg/L	0.16 – 14.8	6.41 ± 1.55	> 5.5^a^
COD mg/L	10.36 – 204.52	62.6 ± 11.1	
BOD mg/L	6.08 – 89.22	29.46 ± 4.89	
NO_3_–N µg/L	35.81 – 931.79	308.75 ± 112.65	2935^a^
NO_2_–N µg/L	ND – 386.4	186.41 ± 51.6	60^a^
NH_4_–N mg/L	0.13–12.27	2.83 ± 1.35	0.077–1.27^a*^
TN µg/L	0.33–18.91	5.02 ± 5.37	
PO_4_^3^–P µg/L	14.30–931.6	188.04 ± 120.1	
TP µg/L	42.41 – 1421.7	408.15 ± 165.4	
Cd µg/L	0.79 – 7.84	2.72 ± 5.1	7.9^b^
Cr µg/L	7.89 – 31.88	15.42 ± 1.18	50^b^
Cu µg/L	3.01 – 25.17	9.69 ± 1.11	3.1^b^
Fe µg/L	149.8 – 831.5	418.21 ± 52	1000^b^
Mn µg/L	34.1 – 97.88	64.69 ± 3.20	100^b^
Ni µg/L	4.62–57.22	26.91 ± 14.45	8.2^b^
Pb µg/L	3.18–23.84	44.52 ± 4.7	8.1^b^
Zn µg/L	30.47 – 91.68	54.97 ± 4.19	81^b^

*Ammonia permissible level dependent on temperature (20–30 °C) and pH value (7.5–8.5), ND = not detected a=^64^, b=^65^.

Lake Manzala exhibited mildly to moderately alkaline conditions, a characteristic feature of Egyptian deltaic lakes where the hydrochemistry is governed by carbonate buffering and intensive photosynthetic activity^32^. The observed spatial pH gradient, with higher values in the central and northern zones, aligns with periods of oxygen supersaturation, suggesting that photosynthetic CO₂ uptake by phytoplankton is a primary driver of alkalinity in these areas. Conversely, the relatively lower pH values near southern drains are likely a signature of microbial respiration and the decomposition of organic-rich inflows, which release organic acids and CO₂^30,63^. The strong positive correlation between pH and DO (*r* = 0.76) further confirms that biogenic processes, rather than mineral inputs, play a pivotal role in regulating the lake’s chemical equilibrium.

Dissolved oxygen (DO) concentrations varied widely between 0.16 and 14.8 mg/L (mean 6.4 ± 1.6 mg/L). Well oxygenated conditions were measured in the northern open‑water areas exposed to wind mixing and seawater exchange, whereas near‑anoxic conditions occurred in southern basins adjacent to Bahr El‑Baqar and other major drains due to the oxidation of organic and ammonium‑rich inflows^32,66^. The notable inverse relationship between DO and BOD (*r* = − 0.38, *p* < 0.01) reflects oxygen depletion caused by microbial oxidation of organic material, a pattern evident in other eutrophic lagoons globally^67,68^. The distribution of BOD_5_ and COD mirrored the lake’s pollution topography, with peak concentrations localized at stations receiving industrial and agricultural effluents from major drains. BOD_5_ values reached a maximum of 89.2 mg/L at southern sites, with an overall mean of 29 mg/L, while COD fluctuated significantly, peaking at 204.5 mg/L (mean = 63 mg/L). The high BOD/COD ratios and their near-linear correlation (*r* = 0.98) point toward a common origin of biodegradable and refractory organic pollutants, highlighting the dominance of untreated wastewater inflows consumption^69,70^. Such persistent organic enrichment not only compromises the lake’s oxidative capacity but also shifts the fish community structure toward more tolerant species, such as *Tilapia zillii*, at the expense of more sensitive indigenous taxa^24^.

Collectively, these parameters delineate a progressive north-to-south deterioration gradient, driven by the massive influx of drainage water. Despite large-scale dredging and restoration efforts, the southern sectors remain ecologically compromised, acting as hotspots for nutrient enrichment and oxygen demand. This confirms that while hydrological connectivity has improved, the ultimate recovery of Lake Manzala’s water quality is contingent upon stringent upstream pollution abatement and the expansion of advanced wastewater treatment facilities^29,32^. Similar gradients in Mediterranean lagoons, such as Ichkeul and Karavasta, suggest that ecosystem resilience in these transitional waters depends heavily on managing the balance between anthropogenic inputs and marine flushing health^71–73^.

### Nutrient salts

**Fig. 2:**
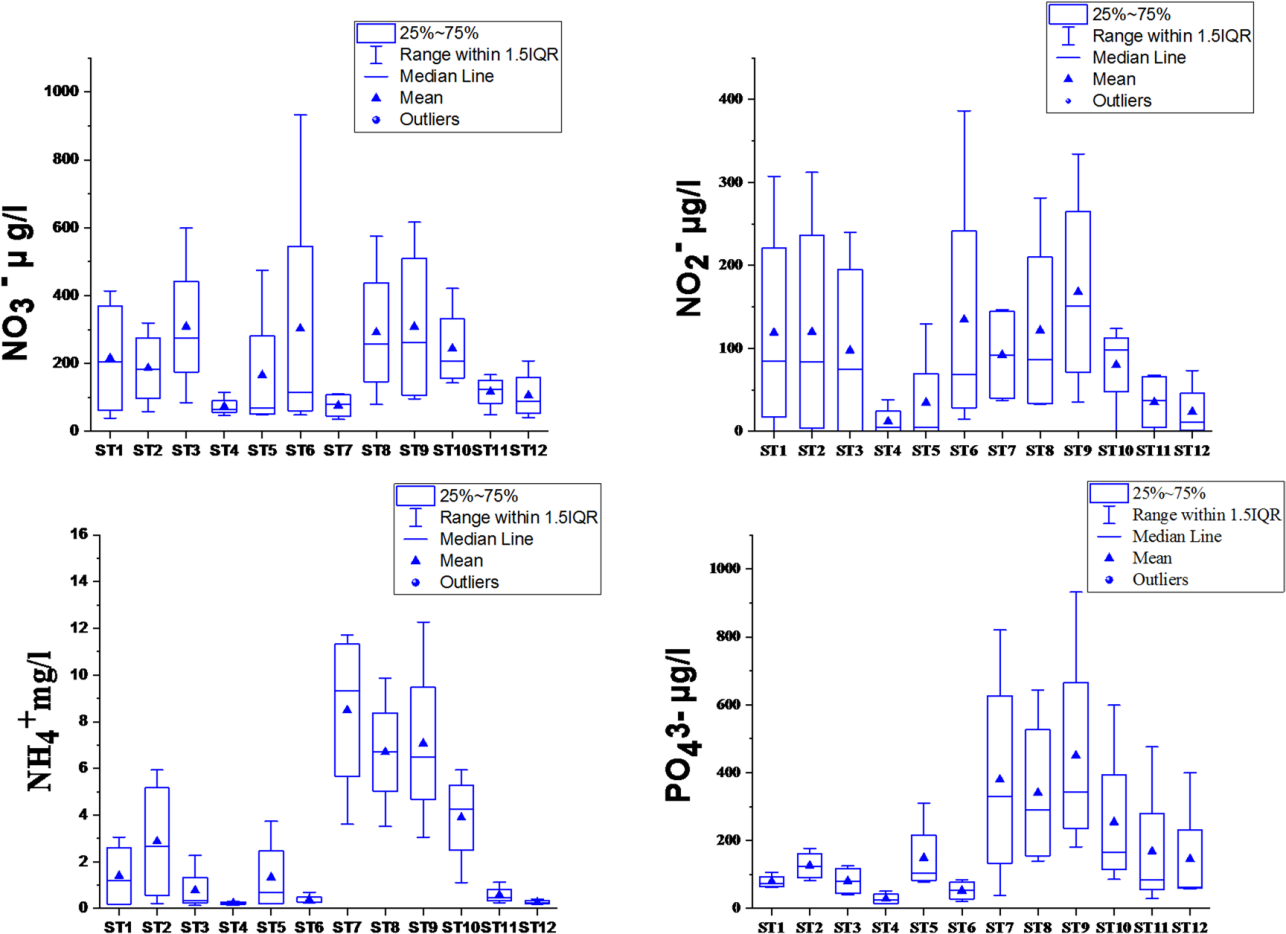
Annual variation of nitrite, nitrate, orthophosphate concentration (µg/L) and ammonia (mg/L) in Lake Manzala during 2021–2022.

Nutrient dynamics within Lake Manzala exhibited pronounced spatial heterogeneity and temporal variability (Fig. 2, Tables S2, S3), mirroring the interplay of anthropogenic inflows, hydrodynamic processes, and biogeochemical transformations. The spatial analysis of nutrient species (Fig. 2) reveals a stark contrast between the lake’s sectors, driven by drainage proximity. Ammonia (NH₃) and phosphate (PO_4_) displayed significant enrichment at stations ST7, ST8, and ST9, which are located near the southern drainage inlets; these stations not only showed the highest median values but also exhibited extensive vertical whiskers, indicating high environmental fluctuations in these pollution hotspots. Conversely, stations ST4, ST11, and ST12, situated near the sea inlets, maintained consistently low and stable concentrations across all species, quantitatively demonstrating the effective dilution and flushing capacity of the newly developed maritime openings. In general, the southern and southeastern basins, directly receiving discharges from the Bahr El-Baqar, Hadous, and Serw drains, consistently displayed the highest nutrient enrichment, whereas the northern and northwestern sectors, influenced by Mediterranean inflow, maintained comparatively lower levels due to dilution and seawater exchange. This spatial pattern clearly highlights the dominance of agricultural and domestic effluents as the main sources of nitrogen (N) and phosphorus (P) enrichment, conditions that typify eutrophic deltaic lagoons^32,63^.

Nitrogen species exhibited distinct seasonal and spatial patterns, reflecting the interplay between agricultural drainage and microbial transformations. Nitrite (NO₂⁻) levels (ranging from BDL to 386 µg/L) peaked during winter, particularly in mid-lake stations. This winter surge is attributed to intensified nitrification under well-oxygenated conditions, where lower temperatures favor the intermediate oxidation of ammonium, a pattern consistent with observations in Lakes Burullus and Edku nitrite^59,63,66^. Conversely, nitrate (NO₃⁻) concentrations reached a spring maximum (up to 931.8 µg/L), specifically at stations influenced by intensive fertilizer runoff. The positive correlation between nitrate and DO (*r* = 0.42) confirms that both microbial nitrification and photosynthetic productivity govern nitrate dynamics. While mean concentrations remained below the CCME acute toxicity guideline (2.93 mg/L), the levels are sufficient to sustain high eutrophication potential throughout the year forms^30,74^. Ammonia concentrations displayed a broad range (0.13–12.27 mg/L), with the most hazardous levels localized in the southern sector near Bahr El-Baqar and Hadous drains. These autumn maxima result from the synergistic effect of heavy organic waste discharge and accelerated bacterial ammonification under warm conditions^32,62^. The inverse relationship between ammonia and DO (*r* = -0.33) underscores the impact of oxygen-demanding decomposition on nitrogen cycling. Such ammonia accumulation in southern basins mirrors conditions in other Mediterranean lagoons like Lesina (Italy) and Ichkeul (Tunisia), where high nutrient inflows coupled with limited hydrodynamic mixing promote nitrogen stratification^71,75^.

Phosphorus dynamics mirrored the spatial variability of nitrogen, with soluble reactive phosphorus (PO_4_–P) ranging from 14.3 to 931.6 µg/L. The spring peak in southern sites (near agricultural drains) suggests a combination of external loading and internal mobilization from sediments via wind-induced resuspension and biochemical regeneration conditions^76–78^. In contrast, northern stations showed a significant decline in phosphorus availability. This trend is likely due to the mixing with high-salinity marine water, which promotes the precipitation of calcium and magnesium phosphates, a well-established geochemical process where increased ionic strength limits orthophosphate solubility^25,79^. Total phosphorus (TP) and total nitrogen (TN) reached maximum values of 1421.7 µg/L and 12.8 mg/L, respectively, confirming a persistent hypereutrophic status. These findings suggest that despite recent hydrological restoration and dredging, the nutrient legacy in the sediments and continuous loading from drainage systems remain significant stressors^62^.

Evaluation of nutrient stoichiometry using the TN: TP molar ratio (ranging from 3.16 to 16.9) revealed a lake-wide nitrogen limitation, particularly in the northern and central basins where ratios fell below 10 (Table 10). In the southern sectors, intermediate ratios (10–17) point toward a potential co-limitation by both nutrients, a condition typical of highly productive, drainage-fed lagoons^80,81^. These relatively low N: P ratios, compared to global averages, indicate a surplus of phosphorus which likely shifts the phytoplankton community toward cyanobacterial dominance during the warmer seasons^63,82^. This stoichiometric imbalance, shared with other Egyptian deltaic wetlands, confirms that anthropogenic loading remains the primary driver of the lake’s ecological shift^25,46^.

**Table 10:** Annual mean N/P ratios in Lake Manzala water (2021–2022).

Station	TN mg/L	TP µg/L	*N*/*P*	Limitation
1	2.618	192.643	13.593	N and P
2	4.716	338.708	13.926	N and P
3	1.891	210.846	8.972	N
4	0.989	97.573	10.1461	N and P
5	6.203	376.398	16.481	N and P
6	1.082	121.2507	8.926	N
7	12.848	756.603	16.981	N and P
8	10.3013	713.865	14.430	N and P
9	11.281	870.193	12.964	N and P
10	6.197	562.734	11.013	N and P
11	1.330	420.284	3.1651	N
12	0.749	236.757	3.166	N

Overall, the enrichment of both nitrogen and phosphorus compounds demonstrates that Lake Manzala remains in a hypereutrophic state, despite ongoing restoration. The persistent influx of drainage-borne organic matter, and untreated wastewater dominates the nutrient budget, resulting in intense algal blooms, severe oxygen depletion, and a distinct spatial gradient that mirrors patterns observed in preceding decades^32,83^. While dredging has improved hydrological flushing, these findings underscore that controlling nutrient inflows at their sources is the most critical requirement for sustainable ecological recovery.

Critically, the same biogeochemical conditions that sustain this hypereutrophic status, high organic matter loading and low benthic oxygen, directly influence the mobility and bioavailability of trace metals. The degradation of organic matter in the southern basins enhances metal release from sediments and promotes the formation of soluble complexes with humic substances. Consequently, the nutrient dynamics in Lake Manzala act as a catalyst for heavy-metal cycling, linking the fate of nutrients to the spatial distribution of metallic contaminants discussed in the subsequent section.

### Heavy metals concentrations

**Fig. 3:**
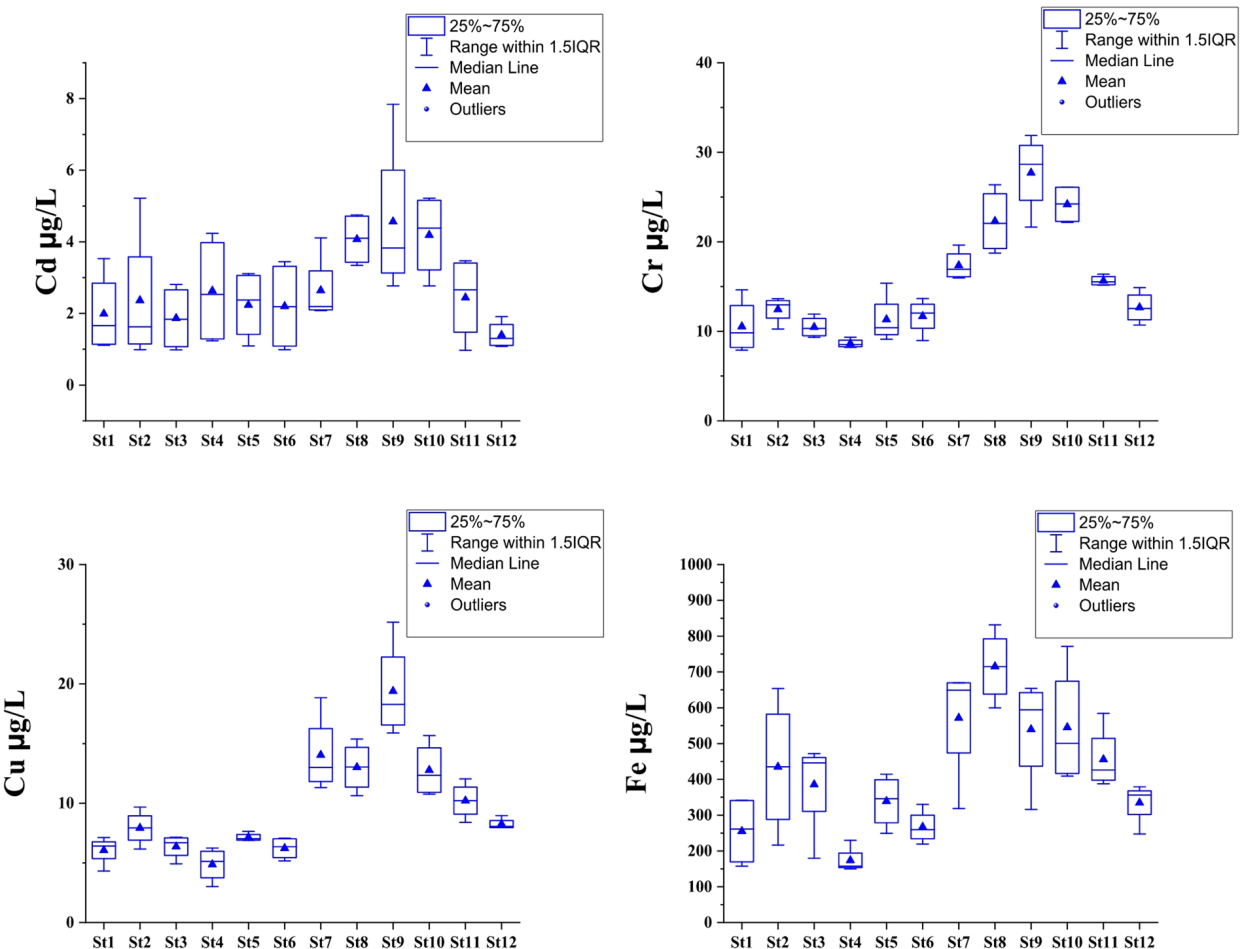
Annual variation of Cd, Cr, Cu, and Fe concentration (µg/L) in Lake Manzala water during 2021–2022.

**Fig. 4:**
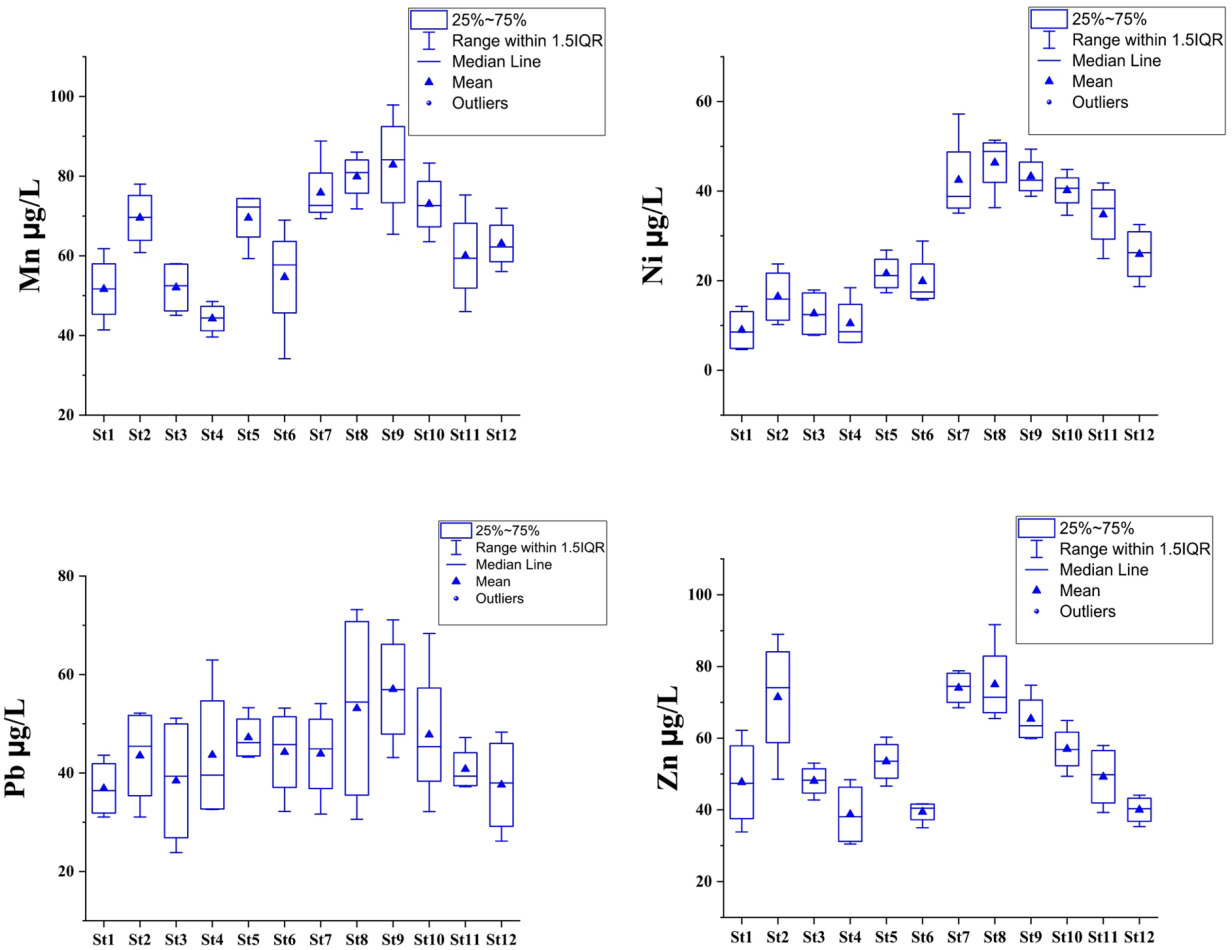
Annual variation of Mn, Ni, Pb, and Zn concentration (µg/L) in Lake Manzala water during 2021–2022.

The concentrations of heavy metals in Lake Manzala demonstrated marked spatial and temporal variations, reflecting the combined influences of anthropogenic discharge, hydrological dynamics, and seasonal changes. The seasonal distribution of heavy metals (Figs. 3 and 4, and Tables S4, S5) reveals a consistent declining trend from autumn 2021 toward summer 2022 across most elements, particularly for Cd, Zn, and Pb. The boxplots highlight that autumn recorded not only the highest median concentrations but also the widest interquartile ranges, indicating greater environmental instability during this period. Conversely, the significantly contracted boxes and lower medians observed in summer (especially for Fe and Mn) quantitatively demonstrate the effect of increased seawater flushing and the potential reduction in external loading toward the end of the study period. On the other hand, the highest concentrations were consistently recorded in the southern and southeastern regions of the lake, particularly near the major drains such as the Bahr El‑Baqar, Hadous, and Serw, which convey large volumes of untreated agricultural, domestic, and industrial effluents. In contrast, the northern and northwestern open‑water areas, which experience better circulation and mixing with Mediterranean seawater, exhibited comparatively lower metal levels. This spatial trend confirms that drainage inflows are the principal sources of heavy‑metal contamination in the lake^30,77,84–86^.

Heavy metal concentrations in Lake Manzala exhibited a consistent spatial and seasonal pattern, with Iron (Fe) being the most abundant (mean = 420 µg/L, max = 831.5 µg/L), followed by Zn, Mn, Pb, Ni, Cr, Cu, and Cd. This sequence (Fe > Zn > Mn > Pb > Ni > Cr > Cu > Cd) mirrors the distribution found in other polluted Mediterranean lagoons where restricted hydrodynamic renewal exacerbates sediment–water interactions^86,87^. Elevated Fe and Mn concentrations reflect the strong influence of suspended soil particles and fertilizer residues carried by agricultural drains, whereas Cu and Zn are mainly derived from domestic effluents, antifouling paints, and the corrosion of metallic structures in nearby urban and industrial areas^88^.

Pb and Cd levels are of critical ecological concern, particularly in the southern sector near the Bahr El-Baqar drain. Pb concentrations (ranging from 24 to 73 µg/L) consistently exceeded international water quality guidelines for aquatic life^65,87^, indicating a high potential for bioaccumulation in the lake’s fisheries. Similarly, Cd peaked at 7.8 µg/L near southern stations, a signature of agricultural runoff enriched with phosphate fertilizer impurities^84,86,88^. The persistence of these toxic elements, despite recent dredging, suggests that they are deeply embedded in the lake’s biogeochemical cycle, posing long-term risks to food security for local consumers^89^.

The observed winter maximum for most metals is primarily driven by the surge in drainage discharge and diminished seawater intrusion. Colder conditions during winter suppress biological uptake and retard the sedimentation of metal-bearing particulates, favoring longer residence times in the water column^90^. Conversely, the summer decline is facilitated by increased salinity (seawater flushing) and intensified biological activity. As evidenced by our pH and DO results, elevated summer photosynthesis promotes the co-precipitation of metals with carbonates and oxyhydroxides, effectively scavenging dissolved metals from the water column into the sediments^91^.

When compared to other major lagoons like Ichkeul (Tunisia), Vembanad (India), and Taihu (China)^71,92,93^, Lake Manzala exhibits equal or higher metallic burdens, confirming its status among the most contaminated transitional wetlands regionally^77,85^. The persistence of Pb, Cd, and Ni at levels exceeding safety thresholds indicates that while physical restoration (dredging) improves flow, it cannot remediate decades of accumulated chemical stress in the sediments^85^. Ecological recovery is thus strictly contingent upon upstream source control and the implementation of advanced treatment for industrial and domestic effluents conveying these toxic loads.

### Water quality and pollution indices

Integrated indices were applied to assess the overall ecological and chemical status of Lake Manzala and to condense the complex physicochemical dataset into concise diagnostic metrics that could be interpreted in terms of aquatic health and pollution intensity. The CWQI, ATI, TSI, PI, and HPI were calculated to provide complementary perspectives representing general water suitability, biological toxicity risk, trophic condition, and trace metal contamination. Together, these indices furnish a comprehensive quantitative overview of the lake’s restoration status following dredging and wastewater management activities.

**Fig. 5:**
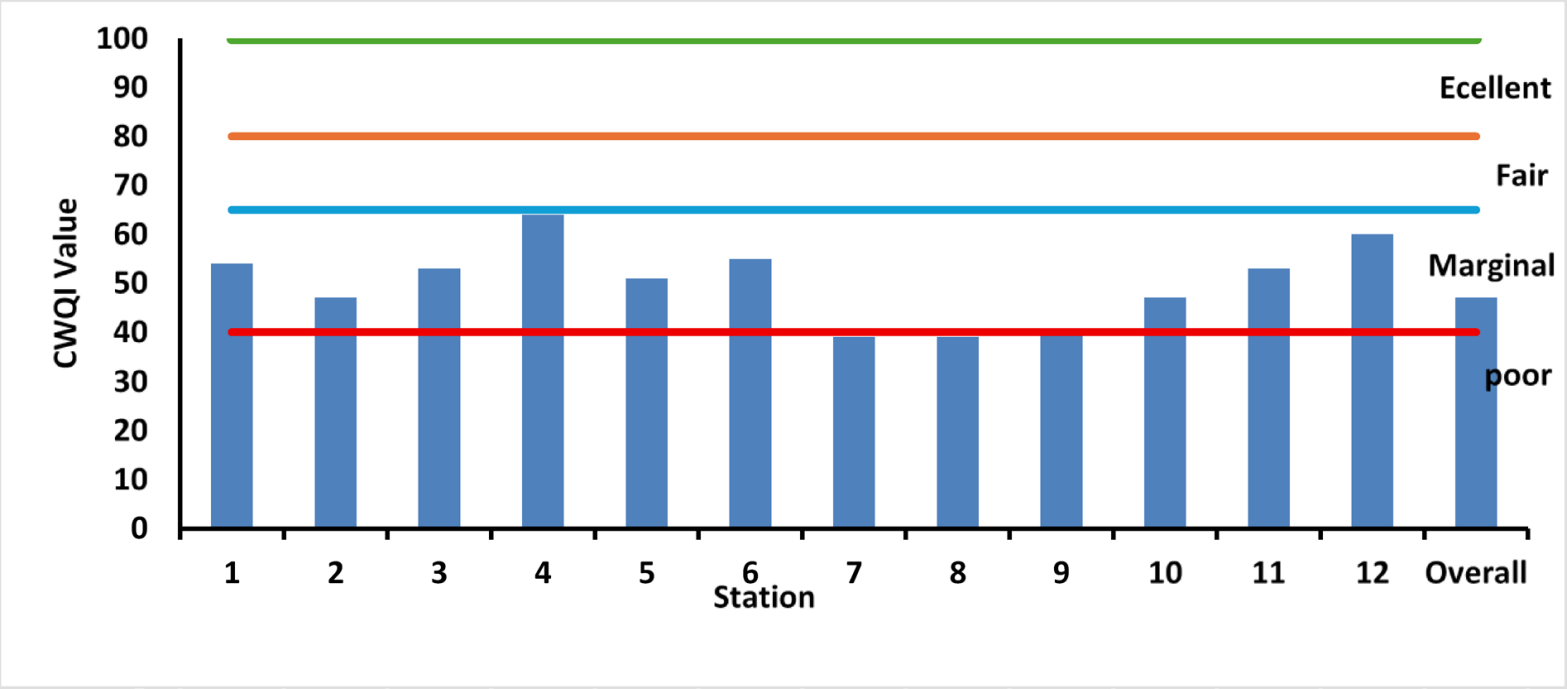
Canadian water quality index of Lake Manzala Water during 2021–2022.

The CWQI scores (Fig. 5) reveal a significant spatial gradient that quantifies the lake’s current environmental status. Scores ranged from a minimum of 39 (poor) at southern stations (e.g., ST7 and ST8) to a maximum of 64 (marginal) at ST4, illustrating a clear shift in water quality as the distance from drainage points increases. The analytical interpretation of Fig. 5 highlights that while the overall average (47) remains in the poor category, a recovery pocket is evident in the open northern and central parts (ST4, ST12), where scores between 60 and 64 suggest a localized benefit from seawater exchange and hydrological circulation improvements. This clearly visualized spatial disparity confirms that the southern basins adjacent to Bahr El-Baqar, Serw, and Hadous drains are the primary drivers of the lake’s ecological stress, consistently falling below the critical 40-point threshold. The overall average CWQI of 47 indicates that Lake Manzala remains ecologically stressed, unsuitable for sensitive aquatic species but potentially tolerable for more resilient taxa. Comparable CWQI ratings were reported for other heavily exploited Egyptian lakes such as Burullus and Mariout^66,94^, underscoring that excessive nutrient loading and limited effective mixing continue to impair the ecological integrity of these deltaic wetlands despite ongoing restoration^32,95^.

**Fig. 6:**
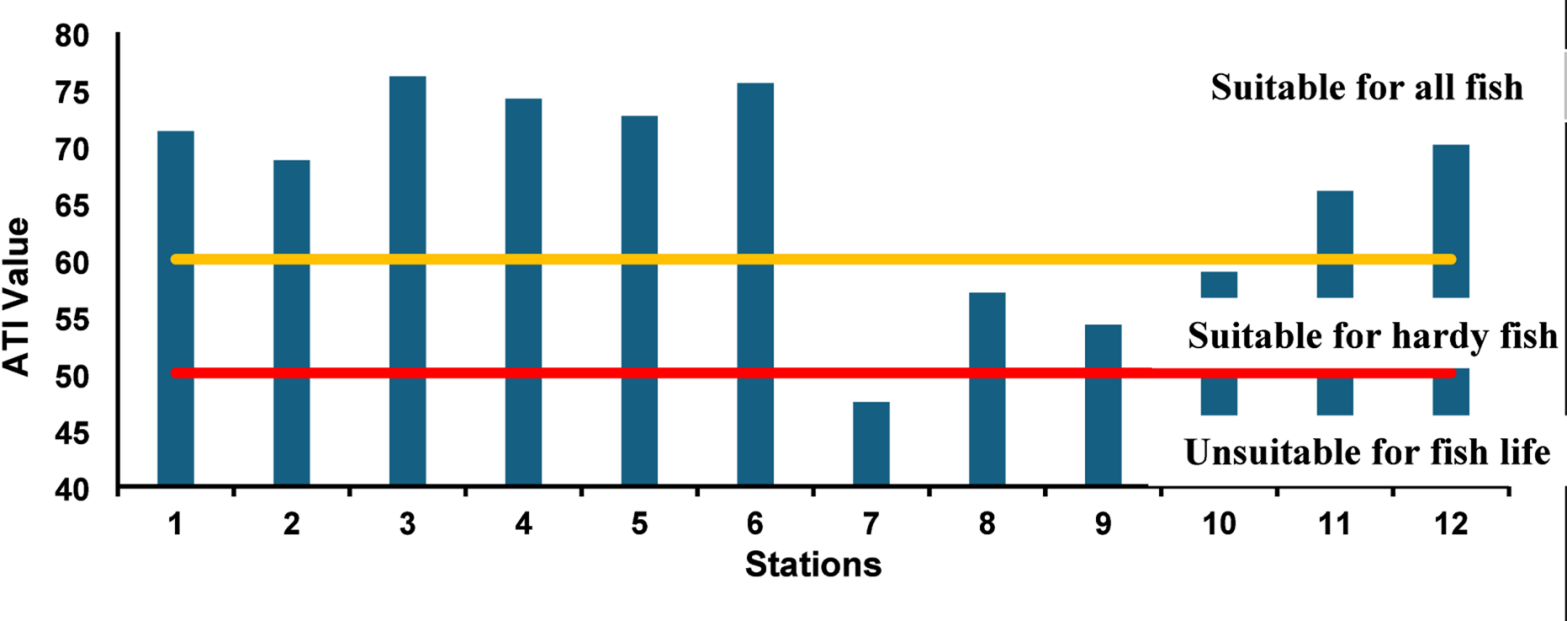
ATI index of Lake Manzala Water during 2021–2022.

The ATI (Fig. 6) provided further insight into the lake’s ability to sustain aquatic life by integrating the combined toxicity effects of dissolved metals and inorganic pollutants. ATI values varied between 44.6 and 86.5, with a critical dip observed at St7, which fell below the threshold line of (50), indicating conditions unsuitable for most fish life. The analytical interpretation of Fig. 6 highlights that while the southern drainage-impacted stations (ST7, ST8, and ST9) remain within the suitable for hardy fish bracket, the northern and central stations (ST3, ST4, and ST6) consistently exceeded the 75-point mark. This spatial heterogeneity, visualized by the varying bar heights against the classification lines, quantifies the vital role of seawater inlets in buffering toxicological stress and creating habitats adequate for sensitive aquatic populations. These patterns corroborate research in other Mediterranean lagoons, where water exchange processes with the sea mitigate toxicity stress^96^. The strong spatial heterogeneity of the ATI suggests that biological sustainability in the lake is governed primarily by proximity to pollution sources rather than by seasonal variation alone.

**Fig. 7:**
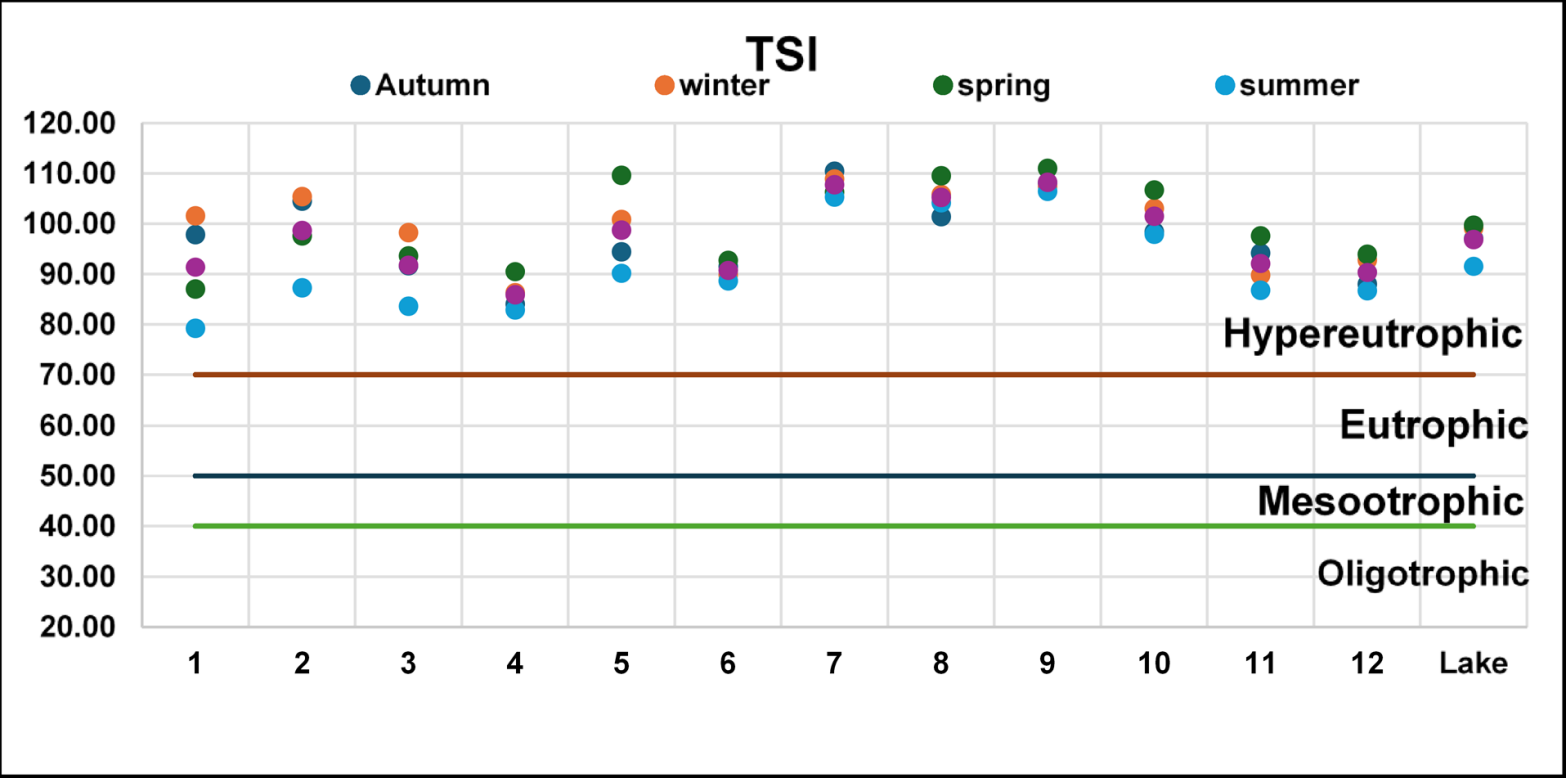
Trophic state index of El-Manzala Lake.

Trophic evaluation based on the Carlson Trophic State Index (TSI) confirmed the lake’s hypereutrophic nature, with index values ranging between 70 and 90 throughout the study (Fig. 7). The highest TSI values (> 85) appeared in the southern and eastern basins exposed to nutrient-rich drain inflows, especially during spring and autumn, marking the periods when biological productivity and nutrient regeneration reach their peaks. Lower but still eutrophic conditions were observed in the northwestern open areas under stronger marine influence. These results are consistent with long-term data^25,63^, which demonstrate that despite recent dredging, Lake Manzala continues to experience excessive nutrient enrichment and algal bloom formation. Comparative analysis with published values from 2004 to 2020^25,32,63,88,97^ confirms that the trophic status has remained consistently in the hypereutrophic domain, indicating chronic nutrient overloading characteristic of anthropogenically impacted lagoons globally^98^.

The Pollution Index (PI) calculated for individual heavy metals revealed that Fe, Mn, Zn, and Cd were within or only slightly above the safe limit (PI < 1), reflecting moderate background levels and partial natural origin. In contrast, Cu, Ni, and particularly Pb showed PI values exceeding 3 at multiple sites (Table 11), implying a strong pollution effect according to^49^. The dominance of Pb related pollution emphasizes its persistence as a problem, consistent with previous findings by^77,84^. The PI spatial pattern reveals distinct pollution hotspots, primarily concentrated near drainage outfalls and urbanized margins.

**Table 11:** PI of Lake Manzala water.

St	Fe	Effect	Mn	effect	Zn	Effect	Cu	Effect	Ni	Effect	Pb	Effect	Cd	Effect
1	0.19	No	0.37	No	0.44	No	1.34	slightly	0.91	No	3.31	strongly	0.26	No
2	0.34	No	0.49	No	0.63	No	1.85	slightly	1.57	slightly	3.75	strongly	0.37	No
3	0.25	No	0.37	No	0.42	No	1.40	slightly	1.19	slightly	3.48	strongly	0.21	No
4	0.14	No	0.31	No	0.35	No	1.12	slightly	1.19	slightly	4.37	strongly	0.31	No
5	0.24	No	0.48	No	0.47	No	1.66	slightly	1.95	slightly	4.23	strongly	0.23	No
6	0.20	No	0.38	No	0.34	No	1.41	slightly	2.05	moderate	3.84	strongly	0.25	No
7	0.37	No	0.56	No	0.64	No	3.54	strongly	4.09	strongly	3.87	strongly	0.32	No
8	0.51	No	0.56	No	0.70	No	3.01	strongly	3.84	strongly	4.90	strongly	0.40	No
9	0.36	No	0.59	No	0.59	No	4.80	strongly	3.83	strongly	5.13	strongly	0.58	No
10	0.44	No	0.52	No	0.50	No	3.07	slightly	3.45	strongly	4.66	strongly	0.41	No
11	0.35	No	0.44	No	0.43	No	2.37	moderate	2.97	moderate	3.71	strongly	0.25	No
12	0.23	No	0.46	No	0.35	No	1.93	moderate	2.29	moderate	3.39	strongly	0.15	No

**Fig. 8:**
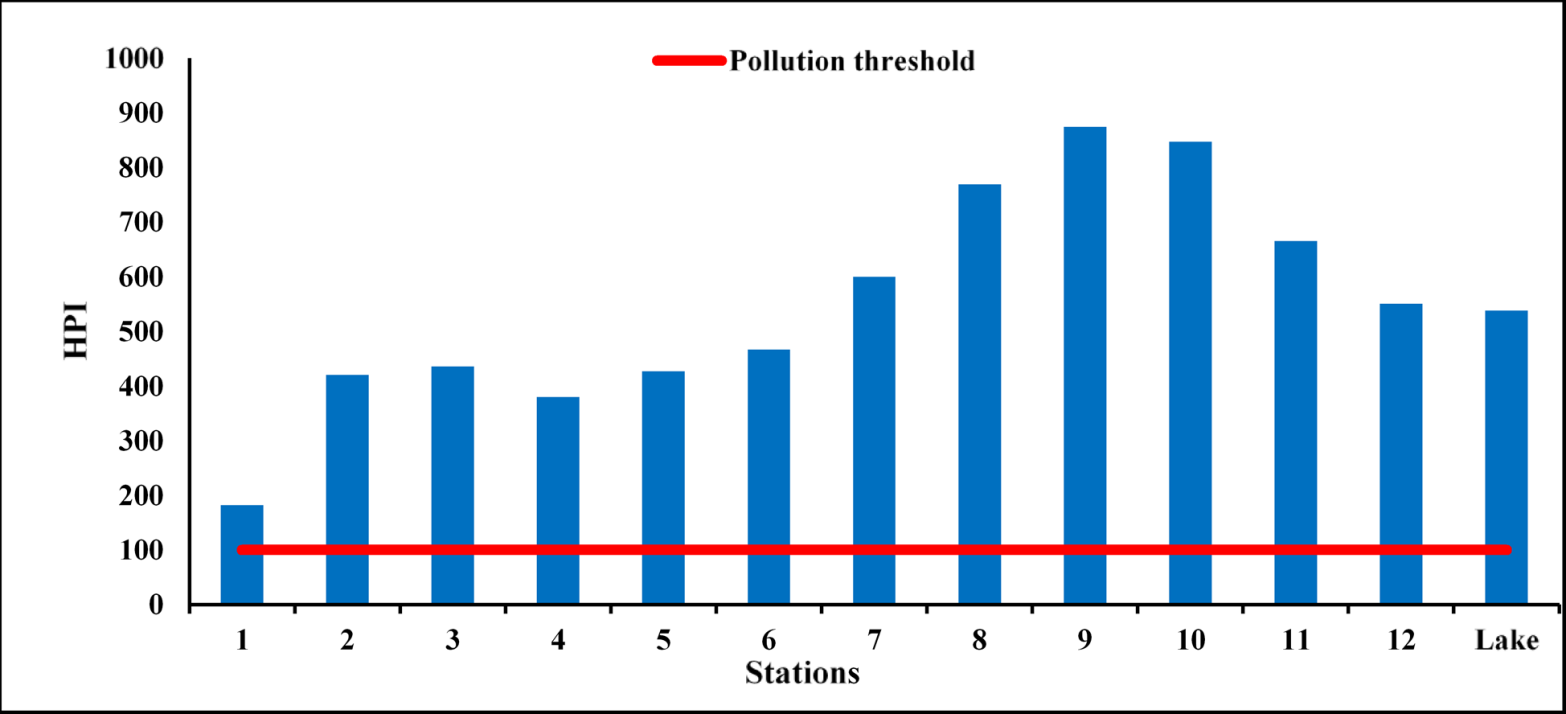
HPI of El-Manzala lake water.

To assess the cumulative influence of trace metals, the HPI was computed. Values ranged between 182 and 874, all markedly surpassing the critical limit of 100, indicating that the lake water is unsuitable for aquatic organisms in sensitive habitats without mitigation. The highest HPI occurred at St9 in the southeastern basin, consistent with the area’s role as the terminal sink of drainage inputs. Although absolute metal concentrations vary seasonally, HPI values consistently categorize Lake Manzala as heavily polluted (Fig. 8). Comparison with national and global shallow lake benchmarks reveals that the lake’s HPI is comparable to, or significantly higher than, those reported for other impacted Egyptian wetlands such as Lake Burullus^15^, Mariout^99^, Edku^100^, Qarun^101^, and Wadi El–Rayan^102,103^. Similar trends are observed internationally in Taihu (China)^11^, and Vembanad (India)^92^, confirming the persistence of intense heavy metal stress in Lake Manzala.

Overall, the combined application of CWQI, ATI, TSI, PI, and HPI indicates that Lake Manzala remains under severe ecological pressure. Although localized improvements in transparency and circulation have been observed after dredging, the obtained data and index integration confirm that the lake’s ecological resilience is still low, with water quality fluctuating between marginal and poor. Sustained environmental monitoring, the reduction of pollutant discharge at the source, and the continued functioning of the Bahr El-Baqar treatment plant are therefore essential for shifting the water quality indices toward fair or good statuses over the long term. Comparable recovery trajectories in northern European and East-Asian lagoons have shown that multi-year consistent treatment and catchment-based nutrient control were prerequisites for measurable WQI improvements^96,104^. The present findings reinforce the conclusion that the successful restoration of Lake Manzala requires an integrated management framework combining hydrological, chemical, and biological monitoring tools within an adaptive long-term strategy.

### Long‑term change in physicochemical and heavy‑metal characteristics of Lake Manzala

Long‑term comparative analysis of Lake Manzala’s water quality over the past four decades (1980–2020 s) (Tables S6 and S7 in the supplementary data) reveals substantial alterations in its hadrochemical regime driven by anthropogenic and climatic pressures. Historical datasets compiled from previous studies^32,62,107–109^  demonstrate that the lake has undergone pronounced shifts in salinity, nutrient loading, and metal concentrations, transforming from a semi‑brackish productive lagoon into a highly eutrophic and metal‑polluted wetland.

During the early 1980s and 1990s, moderate salinity (4–8 psu) and relatively high dissolved oxygen characterized the open basins due to better hydrochemical exchange and hydrological connectivity with the Mediterranean. Since the 2000s, however, salinity has exhibited strong spatial and temporal fluctuations (0.9–40 psu), primarily because of varying drainage inflows, restricted seawater exchange, and extensive sedimentation that reduced average water depth^29^. The dredging operations carried out after 2017 succeeded in partially deepening the lake and re‑establishing connectivity, yet the chemical imprint of decades of eutrophication and industrial contamination persists.

Historical data analysis (Tables S1 and S2) reveals that nutrient loading has been a persistent challenge for Lake Manzala over the last three decades, marked by fluctuating high concentrations rather than a linear trend. While earlier benchmarks^106^ already indicated eutrophic conditions with phosphate levels exceeding 600 µg/L, extreme pollution peaks were recorded in 2017^30^, where values soared above 2000 µg/L. In the present study, although nutrient concentrations remain elevated (NH_4_^+^ up to 11 mg/L; PO_4_^3+^ up to 433 µg/L), they are remarkably lower than the extreme spikes documented in the previous decade, possibly reflecting recent hydrological interventions. Consequently, this excessive nutrient loading has maintained Lake Manzala as a hypereutrophic system typified by persistent algal and macrophyte blooms, reduced transparency, and oxygen depletion^25,26,32^.

Regarding heavy metals, the long-term dataset (Tables S8 and S9) reveals a significant downward trend in metal concentrations compared to the peak pollution years observed between 2008 and 2014. During that critical period, previous studies recorded extreme values, where copper, zinc, and lead concentrations frequently exceeded 500 µg/L^110,111^. In contrast, the present study indicates a substantial reduction in metal loads, with maximum recorded values for Cu, Pb, and Zn remaining below 100 µg/L. Although levels remain higher than those reported in the most recent assessment by^112^, the drastic drop from historical maxima suggests a partial ecological recovery, likely attributed to the recent dredging operations and enhanced water circulation.

Temporal assessments further reveal distinct seasonal patterns. During cold seasons, high wastewater inflow and turbulence enhance nutrient and metal transport, increasing surface concentrations. Conversely, the hot season favors biological uptake and the deposition of particulate metals onto sediments^63,77^. Despite the recent partial improvements, the ecosystem remains enriched relative to original historical background values, confirming that the system is still responding to cumulative catchment-scale anthropogenic impacts. These variations between datasets are likely attributed to differences in sampling strategies (e.g., proximity to drainage outlets versus open water), analytical detection limits, and the specific seasonal timing of collection.

Comparison with other regional wetlands emphasizes a similar pattern of long‑term degradation. Lake Burullus^113^ and Lake Edku^59^ show parallel trends of increasing nutrient loading and metal accumulation, whereas Lake Bardawil, not affected by drainage inflows, has retained good water quality. Hence, Lake Manzala represents an extreme case of lagoonal eutrophication in the southeastern Mediterranean. The combined historical evidence underscores the need for an integrated catchment management policy coupling sediment remediation, continuous monitoring, and stricter upstream control of drainage water to secure long‑term recovery.

### Principal component analysis (PCA)

**Fig. 9:**
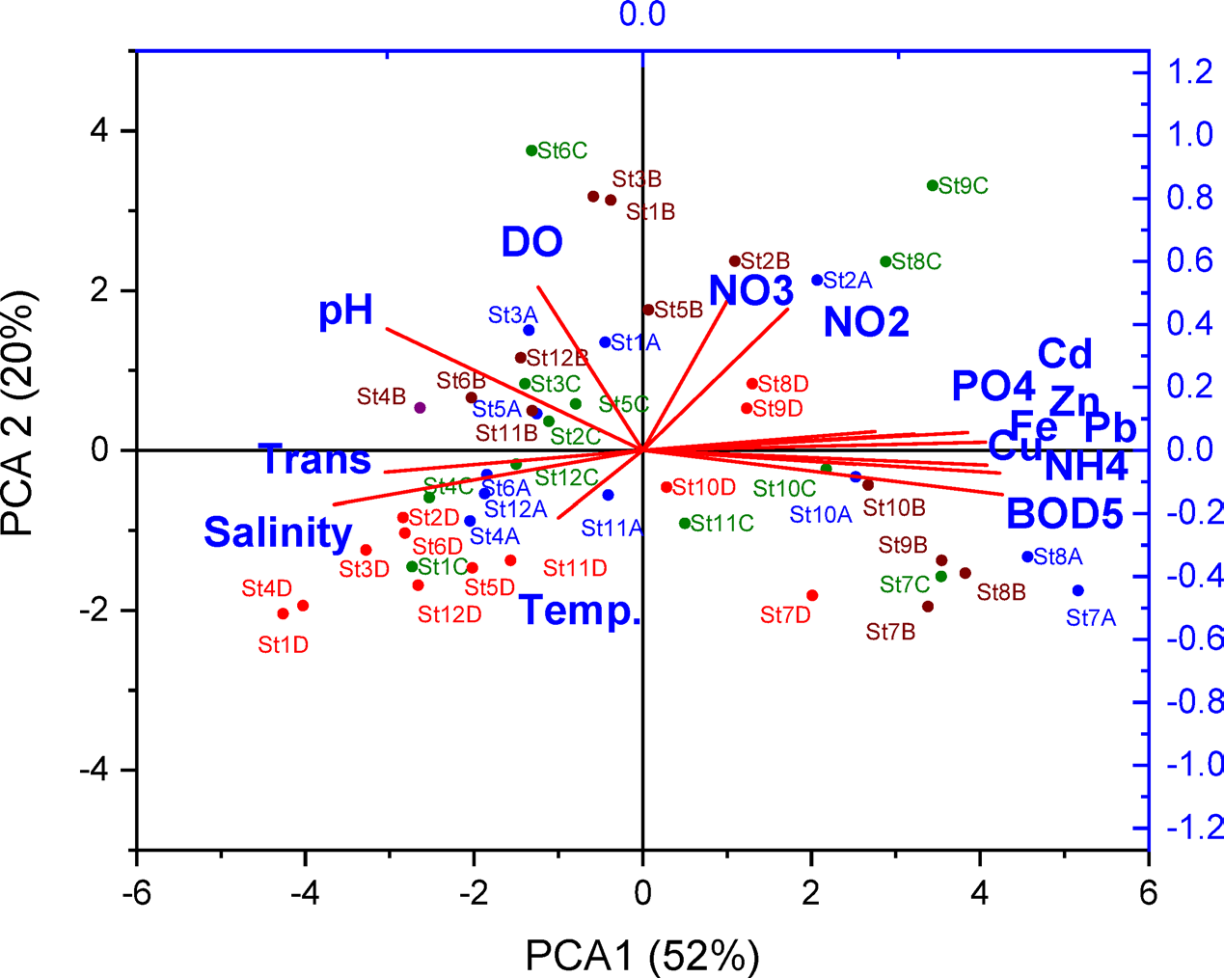
Principal component analysis (PCA) biplot of water quality parameters and sampling stations in Lake Manzala, based on the pooled seasonal dataset (*n* = 48); A= autumn, B= winter, C= Spring, D= summer.

PCA was employed to identify the underlying factors controlling spatial and temporal patterns of water quality in Lake Manzala (Fig. 9). The analysis extracted two principal components explaining about 72% of the total variance. PC1 (about 52%) exhibited strong positive loadings for BOD_5_ (and by extension COD), NH_4_^+^, PO_4_³⁻, and heavy metal variables (Pb, Cd, Zn, Fe, Cu), accompanied by a negative loading for DO. PC2 (about 20%) reflects natural regulatory processes regulating the nitrification cycle and oxygen dynamics, showing positive loadings for NO_3_^−^, NO_2_⁻, and DO, with moderate associations with pH and temperature^114^, a pattern that aligns with the findings of^115^. The PCA biplot distinctly separated the 12 stations into three ecological clusters: (1) a high pollution cluster (St 7–10) driven by PC1, reflecting direct domestic, agricultural, and industrial drainage inflows in the southern/southeastern basins^25,30^. (2) a moderately impacted cluster (St 2, 11, 12); and (3) a relatively clean cluster (St 1, 3–6) grouping along PC2, characterized by higher DO and marine mixing. This pattern matches historical basin-wide analyses and confirms the persistent north–south contamination gradient^25,32^.

Furthermore, seasonal PCA biplots (summer and winter, Fig. S2 in the supplementary file) revealed a consistent pattern of station clustering and variable associations, reinforcing the stability of the observed pollution gradients. In both seasons, the southern and southeastern stations (St7–St10) remained strongly associated with PC1, showing persistent high loadings for heavy metals (Fe, Cu, Zn, and Cd), nutrients (NH_4_^+^, PO_4_^3−^), and organic loads represented by BOD_5_ (and consequently COD). Conversely, the northern open-water stations (St1–St6) consistently grouped with variables such as salinity, transparency, and DO, reflecting the diluting effect of Mediterranean water exchange regardless of the season. The primary seasonal difference was the tighter grouping of NO_3_^−^ and NO_2_^−^ with DO during winter, likely due to enhanced solubility and reduced biological uptake. These seasonal outcomes confirm that while temperature-driven processes (like nitrification and productivity) fluctuate, the anthropogenic-pollution factor remains the overarching driver of Lake Manzala’s water quality throughout the annual cycle.

From a management perspective, the persistent clustering of stations 7–10 on the highly polluted positive axis of PC1 explicitly prioritizes the southern and southeastern sectors for immediate environmental intervention. Rather than basin-wide general measures, future restoration phases must specifically target the primary outfalls driving this cluster—namely, the Bahr El-Baqar and Hadous drains. Implementing advanced wastewater treatment technologies or constructed wetlands directly at the discharge points of these specific drains is the most critical management priority to disrupt the continuous organic and heavy metal loading identified by the PCA.

## Conclusion

Despite recent restoration, Lake Manzala, Egypt’s largest and most economically significant coastal wetland, suffers from severe anthropogenic stress. The 2021–2022 assessment highlights a clear spatial gradient: southern and southeastern basins remain heavily degraded by untreated drainage (Bahr El-Baqar, Hadous, and Serw), whereas northern areas show better quality due to Mediterranean exchange. Analysis confirms hypereutrophic conditions, oxygen depletion, and heavy metal levels (Pb, Cd, Ni, Cr) exceeding international limits. Although dredging improved transparency and salinity, persistent chemical contamination underscores the lake’s limited resilience after decades of pollution. Long‑term comparative analysis showed a clear rising trend in nutrient enrichment and metal accumulation since the 1980s, reflecting cumulative anthropogenic impact despite intermittent remediation. Integrative environmental indices, including the CWQI, ATI, TSI, PI, and HPI consistently categorized Lake Manzala within poor to marginal water quality classes and demonstrated severe heavy‑metal stress (HPI > 100), confirming that the system remains unsuitable for sensitive aquatic life.

In summary, the study highlights that current remediation actions, mainly dredging and wastewater treatment, have been effective in improving hydrological circulation but insufficient to achieve substantial chemical restoration. Ultimately, the transition from a hypereutrophic to a mesotrophic state necessitates a holistic management approach, prioritizing the advanced treatment of agricultural and industrial drainage, specifically from the Bahr El-Baqar system, to safeguard the long-term sustainability of restoration efforts. Therefore, the sustainable rejuvenation of Lake Manzala demands a comprehensive watershed-based framework integrating: (1) complete treatment of all drainage inflows prior to discharge; (2) control of agricultural fertilizer use and industrial effluent release; (3) restoration of natural vegetation belts and establishment of strict protection zones; (4) monitored sediment dredging to prevent pollutant resuspension; (5) continuous environmental monitoring using satellite-based remote sensing coupled with multivariate approaches; and (6) enhanced public awareness and enforcement of environmental regulations. Applying these integrated strategies will not only safeguard the ecological and economic value of Lake Manzala but also serve as a model for restoring other Mediterranean coastal lagoons facing similar pressures in Egypt and globally.

## Supplementary Information

Below is the link to the electronic supplementary material.

Supplementary Material 1

## Data Availability

All data generated or analyzed during this study are included in this published article [and its supplementary information files].
