# Supplementary material for: Evaluating the restoration of Egypt’s Mediterranean Manzala Lagoon: a multi-index assessment of water quality and heavy metals
Source: Sci Rep. 2026 Apr 13;16:12241. doi: 10.1038/s41598-026-45115-x (PMC13076995; doi:10.1038/s41598-026-45115-x)
Supplement: Supplementary file 1 — Supplementary Material 1 [file 41598_2026_45115_MOESM1_ESM.pdf]

Evaluating the Restoration of Egypt's Mediterranean Manzala Lagoon: A Multi-Index Assessment of Water Quality and Heavy Metals

Supplementary Data

Supplementary Text

Water quality indices

S1 Canadian water Quality index

The Canadian Water Quality Index (CCME WQI) [1] provides an effective tool for summarizing complex water quality data into a single, easily interpretable value. This index facilitates communication with stakeholders, policymakers, and the general public by transforming multiple water quality parameters into a standardized score. The CCME WQI is based on three core components: scope (F1), which represents the percentage of parameters that fail to meet water quality guidelines; frequency (F2), which indicates how often the guidelines are exceeded; and amplitude (F3), which reflects the extent to which the guidelines are exceeded. Together, these factors generate an index value ranging from 0 (worst water quality) to 100 (best water quality) (Table 4), which is subsequently categorized into five descriptive classes for ease of interpretation. The three factors are combined to produce a single CWQI value using the following equation:

$$\text{WQI} = 100 - \frac{\sqrt{F1^2 + F2^2 + F3^2}}{1.732}$$

The division by 1.732 normalizes the result to a scale of 0-100, as the maximum length of the vector formed by three factors, each up to 100, is $\sqrt{100^2 + 100^2 + 100^2} = \sqrt{30000} \approx 173.2$.

The specific parameters, guideline thresholds, and assessment periods used in CCME WQI calculations may vary among regions depending on local environmental conditions, intended use of the index, and the water quality concerns. The original CCME User's Manual [41] recommended using a minimum of four parameters measured at least four times annually. Statistical reliability in water quality assessment is significantly enhanced when eight or more parameters are analyzed [13].

S2 Aquatic toxicity index (ATI)

An Aquatic Toxicity Index (ATI) is a specialized type of water quality index specifically designed to assess the potential harm or toxic effects of various contaminants in water bodies on aquatic organisms. Unlike general water quality indices that might focus on overall chemical and physical parameters for various uses (like drinking water or recreation), an ATI is directly concerned with the ecological health of the aquatic ecosystem, often using the response of aquatic biota (like fish, invertebrates, or algae) as indicators. This index was developed by [2]. The modified unweighted additive aggregation function was employed as the aggregation technique. ATI was calculated according to the following equation [3]. The final ATI value ranges from 0 to 100, with higher scores reflecting better ecological water quality and lower toxicity risk. The interpretation of ATI scores follows the classification scheme presented in Table (5), which categorizes water bodies from severely toxic to excellent ecological condition.

The Aquatic Toxicity Index (ATI) is a specialized water quality index designed explicitly to evaluate the potential toxic effects of various contaminants on aquatic organisms. Unlike general water quality indices, which typically focus on the overall physical and chemical suitability of water for human use, irrigation, or recreation, the ATI is directly concerned with the ecological integrity of the aquatic environment. It emphasizes the biological responses of resident

organisms such as fish, invertebrates, and algae, making it a more ecologically relevant indicator in systems where multiple pollutants may interact.

The ATI was originally developed by [2] to provide a quantitative tool capable of integrating the toxicological impact of several contaminants into a single, easy-to-interpret value. This approach is particularly valuable in complex aquatic environments where pollutants often coexist and may exert cumulative or synergistic biological effects that are not adequately captured by evaluating each parameter in isolation.

In the present study, the ATI was calculated using the modified unweighted additive aggregation function, a method that assumes all included toxicants have equal potential to contribute to ecological stress. This non-weighted structure is especially suitable when no single contaminant is known to dominate toxicity, or when the objective is to capture the overall toxic burden of the water body.

The ATI is computed using the following equation [3]:

$$\mathbf{ATI} = \frac{1}{100}\left[\frac{1}{n}\sum_{i=1}^{n} qi\right]^2$$

Where: (q_i) represents the quality rating of the ith parameter, ranging from 0 to 100. Lower values indicate higher potential toxicity, while values closer to 100 reflect negligible toxic effects. The detailed procedure for calculating $q_i$is provided in [42], where each parameter's concentration is evaluated relative to biological tolerance thresholds of standard test organisms. And (n) is the number of determinants included in the index, typically encompassing heavy metals, nutrients, organic pollutants, and other substances known to influence aquatic toxicity.

S3 Trophic State Index (TSI)

The Trophic State Index (TSI) is widely used to evaluate the overall trophic status of lakes by quantifying the degree of nutrient enrichment and the resulting biological productivity. In this study, the trophic condition of Lake Manzala was assessed based on the framework proposed by [4], which integrates the original [5] estimation by incorporating multiple biological and chemical indicators.

Four key variables were used to compute the TSI components: Secchi depth (SD) measured in meters (TSI_SD), chlorophyll-a concentration measured in µg/L (TSI_Chl-a), total phosphorus (TP) measured in µg/L (TSI_TP), and total nitrogen (TN) measured in mg/L (TSI_TN). These parameters collectively reflect water transparency, algal biomass, and nutrient availability, three essential indicators of eutrophication. The trophic status of Lake Manzala was determined using the mean annual values of these individual TSI components. This multi-parameter approach provides an integrated representation of nutrient enrichment and productivity, enabling a robust assessment of Lake Mahzala's ecological condition.

The scoring system for TSI is based on a scale of 0 to 100, where each 10-unit interval represents a specific trophic state. The 'weight' of each parameter is determined by its logarithmic relationship with algal biomass, ensuring that transparency (SD), nutrients (TP, TN), and biomass (Chl-a) are equally reflected in the final mean TSI. To define the performance of each indicator, the raw concentrations were evaluated against the thresholds established by [5,6] as cited in Tables 6a and 6b. The overall index ranges from 0 to 100, where lower values indicate oligotrophic conditions (clear, nutrient-poor water) and higher values indicate eutrophic or hypereutrophic conditions (nutrient-rich, highly productive water).

TSIs calculations were done as in the following equations: -

$\mathbf{TSI_{SD}} = 60 - 14.41 \ln (SD)$
$\mathbf{TSI_{Chl\text{-}a}} = 30.6 + 9.81 \ln (Chl\text{-}a)$
$\mathbf{TSI_{TP}} = 4.15 + 14.42 \ln (TP)$
$\mathbf{TSI_{TN}} = 54.4 + 14.43 \ln (TN)$
$\mathbf{TSI} = 1/4\ [TSI_{SD} + TSI_{Chl\text{-}a} + TSI_{TP} + TSI_{TN}]$

Metal Pollution Indices

S4 Pollution Index (PI)

The PI is one of the most widely used tools for evaluating water quality, particularly in studies focusing on heavy metal contamination. It translates a broad range of environmental measurements into a single, interpretable score that reflects the overall pollution status of a water body [7]. As emphasized by [8], the PI provides an integrated assessment of metal concentrations, allowing for a straightforward classification of pollution levels. The index is calculated for each individual metal, and the resulting values are categorized into five contamination classes (Table 7), ranging from low to severe pollution. According to [9], the Pollution Index is determined using the following equation:

$$Pi=\frac{\sqrt{[\frac{Ci}{Si}]^2{}_{max}+[\frac{Ci}{Si}]^2{}_{min}}}{2}$$

Where: C_i= measured concentration of the heavy metal and S_i= corresponding national or international water quality standard

This formulation allows the index to capture both the minimum and maximum deviations from acceptable limits, offering a more balanced representation of contamination intensity.

S5 Heavy Metal pollution index (HPI)

The HPI is a comprehensive tool developed to evaluate the overall impact of heavy metals on water quality. Rather than analyzing each metal independently, the HPI integrates the collective influence of all measured heavy metals into a single numerical score. It has been widely adopted by environmental agencies because it provides a clear understanding of the cumulative toxic burden in aquatic systems. This index is particularly useful for determining the suitability of water for aquatic life, with a critical threshold value of 100, beyond which the water is considered unsuitable due to heavy metal contamination [10] (Table 8). Originally proposed by [11], the HPI is calculated using the formula.

$$HPI=\frac{\sum_{i=1}^{n} QiWi}{\sum_{i=1}^{n} Wi}$$

Where Qi and Wi represent the sub-index and unit weight of Ith parameter respectively. n is the total number of parameters to be considered.

The sub-index Qi computed as $Qi = \sum_{i=1}^{n} \frac{Mi-Ii}{Si-Ii}\mathbf{100}$

Where Mi and Ii represent the monitored and ideal values of the ith parameter, Si represents the standard value of the ith parameter in parts per million (ppm).

S6 Principal component analysis (PCA)

Principal Component Analysis (PCA) is a multivariate statistical technique widely used to explore patterns within complex environmental datasets and to identify the underlying structure governing variability. The method operates by decomposing the covariance or correlation matrix of the dataset through eigenvalue or singular value decomposition, thereby transforming the original correlated variables into a new set of orthogonal components known as principal components (PCs). Each component represents a linear combination of the original variables and accounts for a progressively smaller fraction of the total variance [12,13].

PCA is particularly advantageous in water quality and environmental studies because it enables the detection of hidden relationships among variables, the grouping of sampling sites based on shared characteristics, and the identification of potential sources of pollution, whether natural (geogenic) or anthropogenic [14,15]. Unlike more restrictive classification methods such as discriminant analysis, PCA does not require assumptions of normality, homoscedasticity, or independence among variables, which makes it a robust and flexible tool for environmental interpretation [16].

In the present study, PCA was applied to simplify and interpret spatial and temporal patterns in the measured water quality parameters. Prior to analysis, the data were standardized (z-score normalization) to eliminate the influence of differing units and magnitudes. Eigenvalues, loading matrices, score plots, and biplots were computed using Origin Pro 2018, which offers advanced multivariate statistical capabilities suitable for environmental datasets. The resulting components were evaluated based on the Kaiser criterion (eigenvalue>1) and cumulative variance explained.

Supplementary Tables

Table S1. Range of values and scoring for TSI indicators based on [45,46]

TSI value	Chl-a (µg/L)	TP (µg/L)	SD (m)	TN (mg/L)	Trophic State	Attributes
0	< 0.04	< 0.75	> 64	< 0.02	**Ultra-oligotrophic**	Excellent
0 – 10	0.04– 0.12	0.75– 1.5	64 – 32	0.02 – 0.09	**Ultra-oligotrophic**	Excellent
10 – 20	0.12 – 0.34	1.5 – 3	32– 16	0.09 – 0.18	**Ultra-oligotrophic**	Excellent
20 – 30	0.34 – 0.94	3– 6	16 – 8	0.18 – 0.37	**Oligotrophic**	Excellent
30 – 40	0.94 – 2.6	6 – 12	8 – 4	0.37 – 0.74	**Oligotrophic**	Good
40 – 50	2.6 – 7.3	12 – 24	4 – 2	0.74 – 1.47	**Mesotrophic**	Fair
50 – 60	7.3 – 20	24 – 48	2 – 1	1.47 – 2.94	**Eutrophic**	Poor
60-70	20 – 56	48 – 96	1 – 0.5	2.94 – 4.93	**Eutrophic**	Very Poor
70-80	56 – 154	96– 192	0.5 – 0.25	4.93 – 5.89	**Hypereutrophic**	Critical
80-90	154 – 427	192– 384	0.25 – 0.12	5.89 – 11.7	**Hypereutrophic**	Highly Degraded
90-100	427 – 1183	384 – 768	0.12– 0.06	11.7 – 23.6	**Hypereutrophic**	Severely Degraded

Table S1. Seasonal ranges and annual means (±SD) of physicochemical parameters across the 12 sampling stations in Lake Manzala (2021–2022).

Station	Temp ^{0}C		Trans cm		EC mS/cm		Salinity PSU		TSS mg/L		pH	
	Range	Range	Range	Mean ±SD	Range	Mean ±SD	Range	Range	Mean ±SD	Range	Range	Mean ±SD
St1	13.3-30.8	20-60	20-60	23.5±18.4	9.9-54.3	32.2±24.6	7.13-38.7	22.2±16.2	58.1-71.9	63.6±6.7	8.3-8.9	8.5±0.33
St2	14.6-30.04	15-35	15-35	23.7±8.5	7.2-31.1	16.9±10.8	5.2-20.1	12.22±7.4	63.9-86.3	74.2±10.1	8.8-9.2	8.9±0.17
St3	13.6-29.7	15-45	15-45	30±12.9	11.9-31.9	18.4±9.1	8.5-20.8	12.5±5.6	43.1-56.2	49.4±5.3	8.6-9.1	8.9±0.22
St4	12.6-28.9	25-60	25-60	42.5±15.5	16.4-43.4	29.5±11.8	11.9-30.3	21±8.2	43.2-62.2	48.9±8.8	8.4-8.9	8.7±0.21
St5	13.1-28.9	15-.50	15-.50	30±14.7	7-25.1	15.2±9.1	5.1-13.7	8.7±4.2	44-82.11	61.3±16.1	8.5-9.2	8.8±0.35
St6	12.7-29.2	25-50	25-50	35±12.2	16.3-30.1	20.7±6.5	10.8-20.3	15.1±4.5	51-78.12	62.3±12.6	8.6-9.1	8.8±0.20
St7	13.2-30.7	5-15	5-15	10±5.7	3.4-3.85	3.7±0.21	2.2-2.7	2.5±0.22	88.3-118	104±12.7	7.4-7.9	7.7±0.23
St8	12.6-31.5	15-30	15-30	21.2±6.3	3.2-4.8	4.15±0.76	2.3-3.2	2.8±0.36	63.8-78.6	70.8±7.7	7.6-8.9	8.2±0.59
St9	13.6-31.2	5-15	5-15	11.2±4.7	3.3-4.01	3.69±0.34	2.3-2.6	2.5±0.17	97.2-126	106±19.7	7.6-8.7	8.1±0.50
St10	12.8-30.8	15-25	15-25	20±5.7	1.66-2.11	1.87±0.22	0.8-1.8	1.4±0.4	48-67.6	57.8±8.6	7.7-8.6	8.1±0.38
St11	13.7-29.2	17-75	17-75	39.2±25.01	1.3-13.06	8.40±5	1.1-8.2	5.6±3.17	45-61.9	53.7±7.1	7.7-8.8	8.4±0.50
St12	13.2-29.1	20-30	20-30	25±5.7	10.2-26.71	17.58±7.16	7.3-19.5	13.7±6.28	49.6-64	53.6±6.9	8.5-9.0	8.7±0.24

Table S2. Seasonal ranges and annual means (±SD) of DO, COD, BOD5, PO_4, and TP across the 12 sampling stations in Lake Manzala (2021–2022).

Station	DO mg/L		BOD_5 mg/L		COD mg/L		PO_4 µg/L		TP mg/L	
	Range	Mean ±SD	Range	Mean ±SD	Range	Mean ±SD	Range	Mean ±SD	Range	Mean ±SD
St1	3.3-13.4	8.0±4.8	6.2-24.5	14.7±9.7	12.1-46.28	27.8±17.4	62.7-106.5	79.3±19.8	89.6-322.8	192±97.8
St2	6.3-14.8	10.8±3.5	13.1-39.7	28.4±11.2	29.5-65.99	50.4±16.27	82.07-177.1	126.3±43.3	145.1-599.1	338.7±192
St3	4.3-12.2	9.4±3.7	10.2-19.8	14.8±4.6	16.5-34.8	25.9±9.17	40.7-125.5	81.1±43.31	121-272	210.8±71.2
St4	2.5-8.5	6.1±2.6	6.1-18.9	9.8±6.1	10.4-34.16	17.3±11.3	14.3-50.6	28.5±16.9	42.4-184.5	97.5±62.8
St5	3.8-14.4	9.3±4.3	19.2-34.6	23.7±7.3	36.1-65.1	44.6±13.72	78.1-310	149.1±109	236.4-518	376±136.3
St6	3.8-9.7	7.1±2.7	8.07-18.5	11.5±4.7	14.06-28.5	18.3±6.8	20.9-84.6	52.7±30.2	76.3-146.8	121.2±32.4
St7	0.16-0.8	0.4±0.2	68.1-89.2	75.5±9.4	163-204	178±18.14	38.5-820.2	379±334.8	583-984	756±190.2
St8	1.2-11.8	5.3±5.1	32.2-74.2	53.7±17.6	86-178.9	132.5±38.9	643.3-139.7	341.1±234	458-931	713±196
St9	0.3-13.2	5.4±6.2	31.6-68.5	48.8±15.4	79.1-158.6	114.6±34.2	181.5-931.6	450±332	473-1421	870±401
St10	1.9-7.1	4.3±2.1	38.4-52.1	42.8±6.3	97.32-102.4	84.5±14.33	85.8-598	254±233	204-1288	562±491
St11	1.5-8.1	4.2±3.02	14.7-20.3	17.5±2.8	28.7-39.16	34.23±4.5	29.2-476	168±206	132-1194	420±516
St12	1.7-9.2	6.2±3.2	10.7-13.7	11.9±1.3	19.8-23.74	22.03±1.74	57.4-399	145.6±169	101-596	236±240

Table S3. Seasonal ranges and annual means (±SD) of nitrogen forms across the 12 sampling stations in Lake Manzala (2021–2022).

Station	NH_4^+ mg/L		NO_3^- µg/L		NO_2^- µg/L		TN mg/L		Ch-a µg/L	
	Range	Mean ±SD	Range	Mean ±SD	Range	Mean ±SD	Range	Mean ±SD	Range	Mean ±SD
St1	0.17-3.05	1.4±1.4	39.9-414	216±181	ND-307	119±137	0.33-6.13	2.62±2.8	98.11-528	320±178.5
St2	0.2-5.9	2.8±2.7	58-319	186±113	ND-312	119±147	0.41-9.22	4.72±4.45	433-720	594±118
St3	0.13-2.2	0.8±1	84-599	308±213	ND-239	97±118	0.6-5.06	1.89±2.13	55.8-407	302±165
St4	0.13-0.2	0.23±0.1	46-115	73±29.5	ND-38.3	12.1±18	0.46-1.87	0.99±0.65	96-382.9	272.5±123
St5	0.2-3.7	1.33±1.6	49-474	165±206	ND-129	34.8±63	0.39-16.18	6.20±7.33	457-796	597±157
St6	0.24-0.7	0.38±0.2	48-931	302±421	14.9-386	134±171	0.62-1.32	1.08±0.31	508-711	582±88.6
St7	3.6-11.7	8.5-±3.6	35-110	76±37.4	37-147	92.2±60	5.48-17.39	12.85±5.42	78.1-281.4	172.8±83.3
St8	3.5-9.8	6.7±2.6	79-574	291±209	32.8-281	121±117	6.42-14.12	10.30±3.16	94.12-682.3	358±242.8
St9	3.1-12.2	7.1±3.8	96-615.8	308±248	35.4-334	168±128	5.27-18.91	11.28±5.65	77.4-636	346±232
St10	1.1-5.9	3.9±2.03	142-421	244±124	ND-124	80.2±54	1.70-9.86	6.20±3.42	217-341.7	294±58.4
St11	0.26-1.1	0.58±0.3	49.5-167	116±49.3	ND-67.6	35.5±35	0.46-2.23	1.33±0.83	176-469	307±129
St12	0.19-0.41	0.26±0.1	41.1-208	106±73.8	ND-73.5	23.7±34	0.38-1.62	0.72±0.588	296-413	384±58.2

Table S4. Seasonal ranges and annual means (±SD) of Cd, Cr, Cu and Fe across the 12 sampling stations in Lake Manzala (2021–2022).

Station	Cd µg/L		Cr µg/L		Cu µg/L		Fe µg/L	
	Range	Mean ±SD	Range	Mean ±SD	Range	Mean ±SD	Range	Mean ±SD
St1	1.1-3.5	1.9±1.1	7.89-14.6	10.5±3.06	4-7	6±1.2	157-341	255±99
St2	0.9-5.2	2.3±1.9	10.26-13.65	12.45±1.51	6-9	7±1.4	216-653	435±188
St3	0.9-2.8	1.8±0.9	9.33-11.92	10.47±1.19	4-7	6±1.02	179-471	385±138
St4	1.2-4.2	2.6±1.5	8.19-9.34	8.64±0.50	3-6	4±1.4	149-229	173±37
St5	1.1-3.1	2.2±0.9	9.11-15.38	11.33±2.77	6-7	7±0.3	249-414	338±74
St6	0.9-3.4	2.2±1.2	8.98-13.66	11.68±1.9	5-7	6±0.9	219-330	267±46.9
St7	2.1-4.1	2.6±0.9	15.96-19.64	17.37±1.68	11-18	14±3.3	318-669	571±169
St8	3.3-4.7	4.1±0.7	18.74-26.37	22.31±3.64	10-15	13±2.1	599-831	715±99
St9	2.8-7.8	4.6±2.2	21.6-31.8	27.7±4.40	15-25	19±4.1	315-654	539±154
St10	2.7-5.2	4.2±1.2	22.16-26.11	24.18±2.21	10-15	12±2.3	408-771	545±168
St11	0.9-3.5	2.4±1.2	15.16-16.38	15.65±0.57	8-12	10±1.5	387-584	455±88
St12	1.1-1.9	1.4±0.4	10.70-14.88	12.67±1.79	7.9-8.9	8±0.47	247-379	334±59

Table S5. Seasonal ranges and annual means (±SD) of Mn, Ni, Pb and Zn across the 12 sampling stations in Lake Manzala (2021–2022).

Station	Mn µg/L		Ni µg/L		Pb µg/L		Cu µg/L	
	Range	Mean ±SD	Range	Mean ±SD	Range	Mean ±SD	Range	Mean ±SD
St1	41-61	51.6±8.5	4.6-14	8.9±4.8	31-43	36±5.6	33-62	47±12
St2	60-77	69.5±7.3	10-23	16±6.3	31-52	43±10.1	48-88	71±17
St3	45-58	52±6.8	7-17	12±5.3	23-51	38±13.6	42-53	48±4.4
St4	39.6-48.5	44.23±3.89	6.2-18	10±5.7	32-62	43±14.3	30-48	83±8.9
St5	59.3-74.38	69.5±7.11	17-26	21±4.1	43-53	47±4.7	46-60	53±5.9
St6	34.16-68.9	54.6±14.6	15-28	19±6.1	32-53	44±9.3	35-41	39±3.1
St7	69.3-88.8	75.8±8.77	35-57	42±10.1	31-54	43±9.5	68-78	74±4.8
St8	71.78-86.04	79.8±6.01	36-51	46±6.8	30-73	53±20.8	65-91	74±11
St9	65.41-97.88	82.88±13.53	38-49	43±4.4	43-71	57±11.9	59-74	65±6.9
St10	63.51-83.28	72.99±8.18	34-44	40±4.2	32-68	47±15.1	49-64	56±6.4
St11	45.97-75.30	60.02±12.066	24-41	34.7±7	37-47	40±4.5	39-57	49±8.8
St12	56.05-71.9	63.10±6.6	18-32	25±6.1	26-48	37±10.2	35-44	40±3.9

Table S6. Long term change in physicochemical characteristics of El-Manzala Lake water during winter.

Year	Temp (°C)	Salinity (‰)	EC ms/cm	pH	NO_3 (µg/L)	NO_2 (µg/L)	NH_4 (mg/L)	PO_4 (µg/L)	DO (mg/L)	BOD (mg/L)	COD (mg/L)	Reference
1993	20.7± 8.3			*8.2 ±0.6					*6.85 ±6.8	*3.66 ±3.2	*26.0 ±16.6	Badawy et al.,1995 [17]
*1995		2.71-39.1		7.6-8.8					*(ND-13.7)			Dewidar and Khedr, 2001 [18]
1999-2000	17.6-24			6.8-7.67	262.8-821	4-185	2.4-9.31	284-687.5	0.5-5.9	1.4-12.1	3.2-20.8	Abdel-Satar, 2001 [9]
2000-2001	16-18			7.41-8.09					4.40-10	3-7	6.80-15.20	Elewa et al., 2007 [20]
2001-2002	11-13	1.23-2.41		8.3-8.8	42.7-404.2	5.39-116.39	0.007-0.68	2.7-106.44	7.5 - 9.3			Shakweer (2006) [21]
*2004		*1.1-22.5		*7.45-8.90	*26.7	*221.6	*0.196-7.23	*101-980	10.2		10	Ali, 2008 [22]
2007	12.4	1.77		8.6	249	53.15	0.009	40.5	8.4			Shaltout and Galal,2007 [23]
2008-2009	14-17	1.49-36.3		7.14-9.18	47.9-245	2.35-50.97	0.05-2.65	34-806	0.8-8.30		4.60-21.1	Abdel-Rasheed, 2011 [24]
*2008-2009	*7.9-9		0.0029-0.018									Hamed et al., 2013 [25]

**2010-2011**	14-16		2.35- 8.10	7.74-8.64	1.25-2.22	25.69-50.76			1.5-5.18		4.6-17.6	Abdel Mola and Shehata,2012 [26]
**2014**		2.2-29.5		7.7-8.6	30-430	20-320	1-6.6	140-390	2.4-6.7			Abu-Khatita etal.,2017 [27]
**2015**	14	1.1 -19.8	2.02-29.2	7.70 – 8.7	46.2-343.2	36.5 – 193.2	0.088 - 1.14	95.1–265.5	1.6 – 11.8	5.76 -56.8	-	El-Morsy et al., 2017 [28]
**2015-2016**	12.4-14.7	1.02-25.2		7.92-8.59					ND-12.1			Goher et al., 2017 [29]
**2017**	13.5-14	1.03-43.4		8-9.2	70-7880	60-760	2.8-32.3	260-2050	3.4-12.8			Ismail and Hettiarachchi2, 2017 [30]
**2017**	14.3-17			7.7-8.7					2.8-11.7	6.8-8.9	30.5-.95.0	Beheary et al.,2019 [31]
***2018**	15	14		7.55-8.82								Orabi et al., 2019 [32]
**2020**	15.6-16.9	1.39-8.89		7.62 -8.97	112.11-850.36	53.33 - 273.96	0.47-14.62	24.20-113.30	0.7-14.06	8.34-76.80		Mahmoud et al.,2022 [33]
**Present Study**	12.61-14.60	1.51-11.94		7.40 -8.98	53.36-599.11	34.19-312.12	0.129-10.94	16.30-432.86	0.16-13.43	6.19-68.16	11.08-169.86	Present study

Table S7. Long term change in physicochemical characteristics of El-Manzala lake water during summer

Year	Temp (°C)	Salinity (‰)	EC ms/cm	pH	NO_3 (µg/L)	NO_2 (µg/L)	NH_4 (mg/L)	PO_4 (µg/L)	DO (mg/L)	BOD (mg/L)	COD (mg/L)	Reference
**1993**	20.7± 8.3			*8.2 ±0.6					*6.85 ±6.8	*3.66 ±3.2	*26.0 ±16.6	Badawy et al.,1995 [17]
**1995**		2.71-39.1		7.6-8.8					*(ND-13.7)			Dewidar and Khedr, 2001 [18]
**1999-2001**	25-30		2.61-6.56	7.1-7.9	1.7-70.2	22-469	2.55-12.19	303.3-1157	ND-4.6	4.5-10.9	4-19.8	Abdel-Satar, 2001 [19]
**2000-2001**	25-30		0.0003-0.054	7.18-8.64					3-5.80	4.20-8.40	8-19.40	Elewa et al., 2007 [20]
**2001-2002**	27.5-30	1.91-4.64		7.8-8.5	7.126-143.7	ND-114.6	ND-0.035	4.59-44.95	7.1-10.7			Shakweer (2005) [21]
***2004**		*1.1-22.5		*7.45-8.90	661	*3.2-221.6	*0.196-7.23	*101-980	ND		31.2	Ali, 2008 [22]
**2007**	29.1	2.7		8.2	75.6	16.8	0.14	18	8.3			Shaltout and Galal,2007 [23]
**2008-2009**	28-32	1.56-36.77		6.98-8.95	64.7-532.1	4.56-76.42	0.27-2.11	115-618	ND-5.10		5.9-30.75	Abdel-Rasheed ,2011 [24]
***2008-2009**	*7.9-9		0.0029-0.018									Hamed et al., 2013 [25]
**2010-2011**	31-32		2.47-8.21	7.67-8.21	0.09-3.27	7.24-89.45			2.20-5.10		5.9-19.4	Abdel Mola and Shehata,2012 [26]
**2014**		1.2 – 31.5		8.1-9.5	40-550	30 - 340	1.3-8.1	100-360	1.2-5.5			Abu-Khatita etal.,2017 [27]
**2015**	25-27.5	1 - 4.6	1.78-7.90	7.76-8.52	8.96-39.76	59.5-211	0.082-3.47	51.7-341.7	1.25-8	5.20-52.8	-	El-Morsy et al., 2017 [28]
**2015-2016**	24-26.8	0.70-24.8		7.28-8.95					0.39-8.86			Goher et al., 2017 [29]

**2017**	30.5-31	1.48-40.8		7.8-8	10-7190	ND-680	2.5-29.1	940-2370	2.5-10.2			Ismail and Hettiarachchi2, 2017 [30]
**2017**	27-35.5			7.9-9					4.9-9.8	7.4-9.6	24.1-168.9	Beheary et al.,2019 [31]
**2018**	33.5	32		7.55-8.82								Orabi et al., 2019 [32]
**2020**	29.22-32.45	1.17-21.9		7.56 -8.93	63.75-705.00	19.74 - 125.16	0.10 - 10.94	9.90 - 143.00	ND-14.70	8.25 - 88.60		Mahmoud etal.,2022 [33]
**Present study**	28.94-31.46	0.89-38.73		7.90-8.90	35.81-405.10	ND-281.40	0.211-7.70	38.50289.30	0.81-7.77	6.08-70.59	10.36-163.27	Present study

Table S8. Long term change in heavy meatal concentration of El-Manzala Lake water during winter

**Year**	**Fe(mg/L)**	**Mn(µg/L)**	**Cu(µg/L)**	**Cr(µg/L)**	**Cd(µg/L)**	**Pb(µg/L)**	**Ni(µg/L)**	**Zn(µg/L)**	**Reference**
**1999-2000**	2.621-5.75	100.5-218.6	8.18-19.36			36.29-61.12		31.30-26.14	AbdelSatar, 2001 [19]
**2000-2001**	0.71-4.80	24-179	9-20		19-34	ND-50		ND-23	Elwa et al., 2007 [20]
**2001-2002**			9-53		ND-21	ND-12		139-232	Bahnasawy et al., 2011 [34]
**2004**	0.477-970	350-790	6.1-11		2.5-4.3	29.7-55		65-93	Ali, 2008 [22
**2007**	*0.72-1.98	280-840	360-680		10-90	12-99		320-660	Saeed and Shaker, 2008 [35]
**2008-2009**	*	720	150		57	230		589	Hamed et al., 2013 [25]
**2008-2009**	1.72-4.18	188-343	17-61			72-87		10-105	Abdel-Rasheed, 2011 [24]
**2012**	1.04-2.97	610-900	430-660		43-86	230-420		540-670	El-Saharty ,2014 [36]
**2015-2016**	0.251-0.862	11.29-34.62	4.78-14.95		1.04-3.68	14-74.66		26.32-58.22	Goher et al., 2017 [29]
**2017**	0.01-0.56		40-850				ND-2	ND-290	Ismail and Hettiarachchi2, 2017 [14]
**2020-2021**	0.03-0.11	ND-20	2-18	1-9	1-2	ND	2-32	6-22	Al-Falal et al., 2024 [37]
**2021-2022**	0.157-0.753	34.16-81.24	3.01-15	7.89-27.63	1.47-5.22	31.06-73.18	10.98-57.22	31.9-91.6	Present Study

Table S9. Long term change in heavy meatal concentration of El-Manzala Lake water during summer

Year	Fe (mg/L)	Mn (µg/L)	Cu (µg/L)	Cr (µg/L)	Cd (µg/L)	Pb (µg/L)	Ni (µg/L)	Zn (µg/L)	Reference
**1999-2000**	0.617-2.804	135.3-441.4	10.63-20.20			29.3-70.52		11.64-27.86	AbdelSatar, 2001 [19]
**2000-2001**	0.09-0.84	28-395	11-22		10-25	28-57		10-27	Elwa et al., 2007 [20]
**2001-2002**			31-192		19-38	17-74		301-529	Bahnasawy et al., 2011 [34]
**2004**	0.750-1.212	490-920	4.3-8.7		3.2-5.6	43.6-71.5		36.6-72	Ali, 2008 [22]
**2007**	*0.72-1.98	280-840	360-680		10-90	12-99		320-660	Saeed and Shaker, 2008 [35]
**2008-2009**	*	720	150		57	230		589	Hamed et al., 2013 [25]
**2008-2009**	1.77-4.28	67-324	11-28			25-42		5-62	Rasheed, 2011 [24]
**2012**	1.12-2.56	540-910	410-640		58-70	340-560		510-670	El-Saharty ,2014 [36]
**2015-2016**	0.216-0.736	8.65-26.19	4.25-14.29		0.98-3.18	7.95-65.5		22.18-56.25	Goher et al., 2017 [29]
**2017**	0.32-0.63		10-410				ND-2	70-350	Ismail and Hettiarachchi2, 2017 [30]
**2020-2021**	0.03-0.11	ND-20	2-18	1-9	1-2	ND	2-32	6-22	Al-Falal et al., 2024 [37]
**2021-2022**	0.157-0.599	41.38-79.68	4.31-17.23	8.51-26.37	0.98-3.52	26.16-47.76	4.63-47.6	30.4-71.5	Present Study

Supplementary Figures

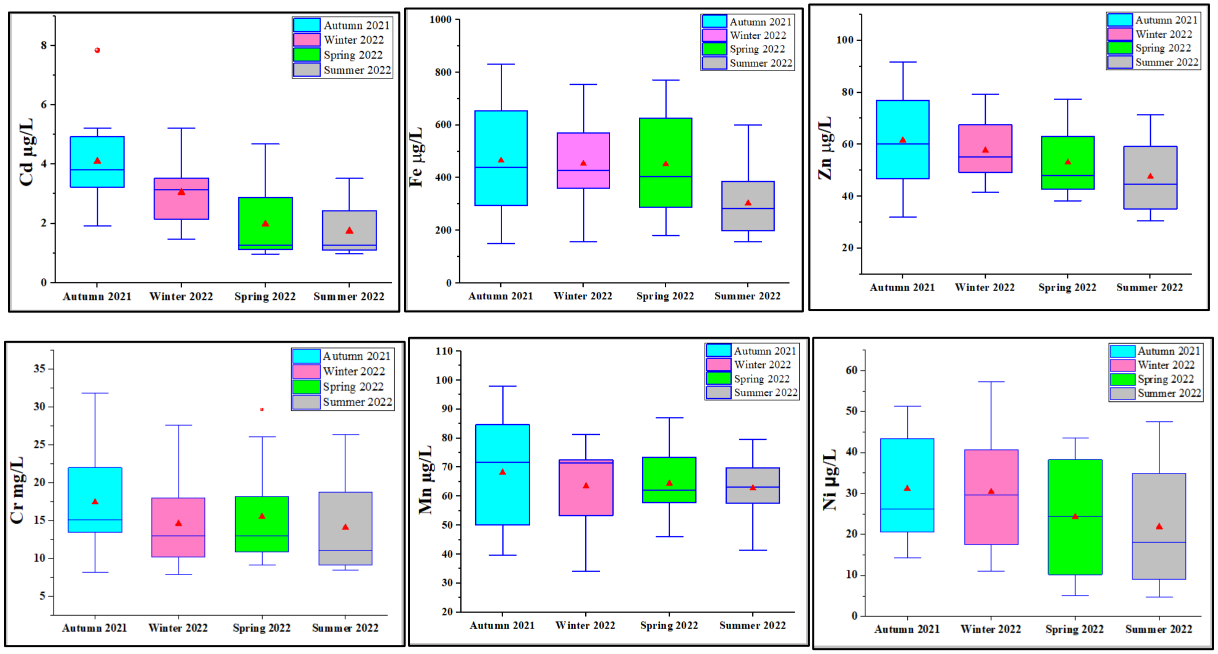

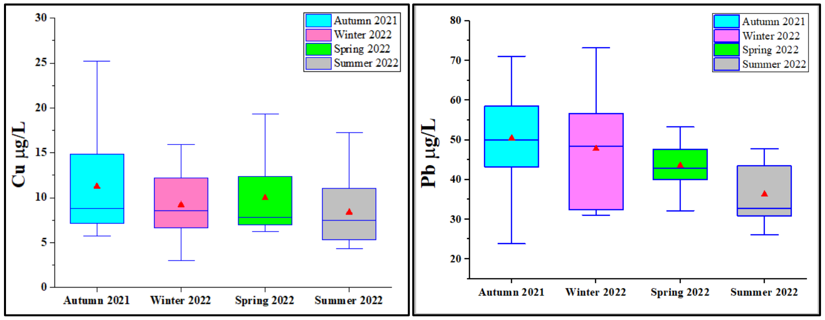

Fig. S1. Seasonal variation of heavy metal (µg/L) in El-Manzala Lake water during (2021-2022)

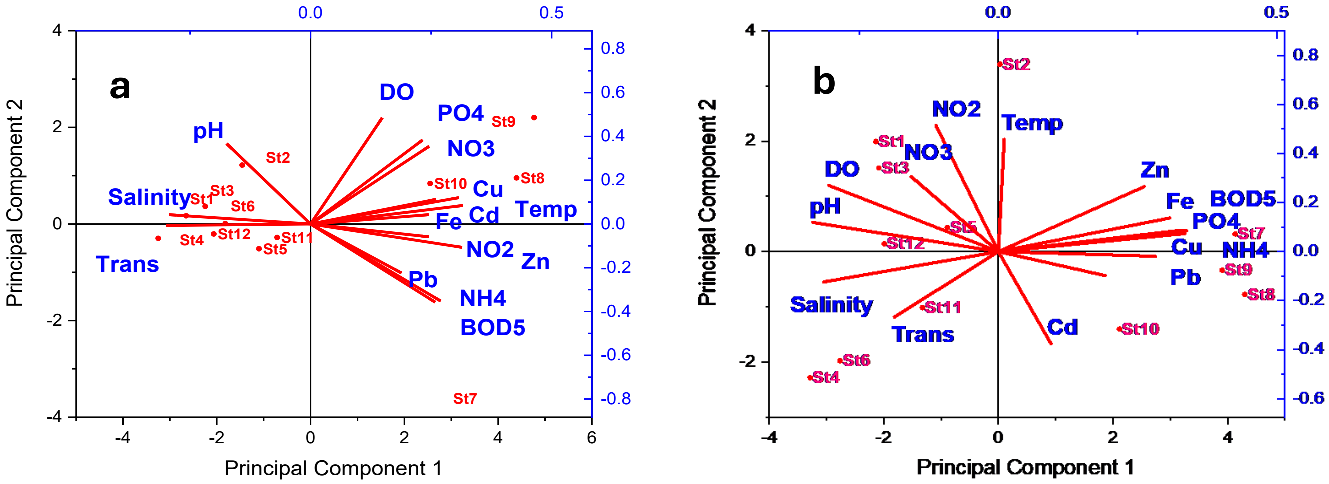

Fig. S2 Principal component analysis (PCA) ordination of sampling stations based on parameters studied during a (summer) and b (winter).

References

1. CCME. Canadian water quality guidelines for the protection of aquatic life: CCME Water Quality Index User's Manual 2017 Update. (2017).
2. Wepener, V., Euler, N., Van Vuren, J. H. J., Du Preez, H. H. & Kohler, A. The development of an aquatic toxicity index as a tool in the operational management of water quality in the Olifants River (Kruger National Park). *Koedoe* **35**, 1–9 (1992).
3. Gupta, S. & Gupta, S. K. A critical review on water quality index tool: Genesis, evolution and future directions. *Ecol. Inform.* **63**, 101299 (2021).
4. Carlson, R. E. & Simpson, J. *A Coordinator's Guide to Volunteer Lake Monitoring Methods*. (North American Lake Management Society, 1996).
5. Carlson, R. E. Atrophic state index for lakes. *Limnology Oceanography* **22**, 361–369 (1977).
6. Kratzer, C. R. & Brezonik, P. L. A Carlson-type trophic state index for nitrogen in Florida Lakes. *Water Resources Bulletin* **17**, 713–715 (1981).
7. Dunca, A. M. Water pollution and water quality assessment of major transboundary rivers from Banat (Romania). *J. Chem.* **2018**, 1–8 (2018).
8. Popović, N. *et al.* Application of the water pollution index in the assessment of the ecological status of rivers: a case study of the Sava River, Serbia. *Acta Zool. Bulg.* **68**, 97–102 (2016).
9. Caeiro, S. *et al.* Assessing heavy metal contamination in Sado Estuary sediment: An index analysis approach. *Ecol. Indic.* **5**, 151–169 (2005).
10. Milivojević, J., Krstić, D., Šmit, B. & Djekić, V. Assessment of heavy metal contamination and calculation of its pollution index for Uglješnica River, Serbia. *Bull. Environ. Contam. Toxicol.* **97**, 737–742 (2016).
11. Mohan, S. V, Nithila, P. & Reddy, S. J. Estimation of heavy metals in drinking water and development of heavy metal pollution index. *Journal of Environmental Science and Health, Part A* **31**, 283–289 (1996).
12. Jolliffe, I. T. & Cadima, J. Principal component analysis: a review and recent developments. *Philosophical Transactions of the Royal Society A* **374**, 20150202 (2016).
13. Jackson, J. E. *A User's Guide to Principal Components*. (Wiley, New York, 2005).
14. Shrestha, S. & Kazama, F. Assessment of surface water quality using multivariate statistical techniques: A case study of the Fuji river basin, Japan. *Environmental Modelling & Software* **22**, 464–475 (2007).
15. Vega, M., Pardo, R., Barrado, E. & Deban, L. Assessment of seasonal and polluting effects on the quality of river water by exploratory data analysis. *Water Res.* **32**, 3581–3592 (1998).
16. Parus, P. Urban water quality evaluation using multivariate analysis. *Acta Montanistica Slovaca* **12**, 150–158 (2007).
17. Badawy, M. I., Wahaab, R. A. & Abou Waly, H. F. Petroleum and chlorinated hydrocarbons in water from Lake Manzala and associated canals. *Bull Environ Contam Toxicol* 55, (1995).
18. Dewidar, K.H. & Khedr, A. Water quality assessment with simultaneous Landsat-5 TM at Manzala Lagoon, Egypt, Hydrobiologia, 457, pp. 49–58 (2001).
19. Abdel-Satar, A. M. Nutrient status and speciation of Lake El-Manzalah sediment. Egypt J Aquat Biol Fish 5, 263–282 (2001).
20. Elewa, A.A., Saad, E.A., Shehata, M.B. & Ghallab, M.H. Studies on the effect of drain effluents on the water quality of Lake Manzala, Egypt, Egyptian Journal of Aquatic Biology and Fisheries, 11(2), pp. 65–78 (2007).
21. Shakweer, L.M. Ecological and fisheries development of Lake Manzalah (Egypt): Hydrography and chemistry of Lake Manzalah, Egyptian Journal of Aquatic Research, 31, pp. 251–270 (2006).
22. Ali, M. H. H. Assessment of some water quality characteristics and determination of some heavy metals in Lake Manzala, Egypt. Egypt J Aquat Biol Fish 12, 133–154 (2008).
23. Shaltout, K.H. & Galal, T.M., Ecosystem of Lake Manzala, https://www.researchgate.net/publication/280597558, DOI:10.13140/RG.2.1.2044.1448.(2011)
24. Abdel Rasheed, M. El-S., ecological studies on lake el-manzalah with special reference to their water quality and sediment productivity, Ms.c Al-Azhar University Faculty of Science Zoology department.(2011) .

25. Hamed, Y.A., Abdelmoneim, T.S., ElKiki, M.H., Hassan, M.A. & Berndtsson, R. Assessment of heavy metals pollution and microbial contamination in water, sediments and fish of Lake Manzala, Egypt, Life Science Journal, 10 (1), pp. 86–99 (2013).
26. Abdel Mola, R. H. &Shehata, M. Effect of drains on the distribution of zooplankton at the southeastern part of Lake Manzala, Egypt, Egypt. J. Aquat. Biol. & Fish., Vol. 16, No. 4: 57-68 (2012).
27. Abu-Khatita, A.M., Shaker, I.M. & Shetaia, S.A. Water quality assessment and potential health risk of Manzala Lake, Egypt, Al-Azhar Bulletin of Science, 9th Conf., pp. 119–136 (2017).
28. Elmorsi, R. R., Hamed, M. A. & Abou-El-Sherbini, K. S. Physicochemical properties of Manzala Lake, Egypt. Egypt J Chem 60, 519–535 (2017).
29. Goher, M. E., Abdo, M. H., Bayoumy, W. A. & Mansour El-Ashkar, T. Y. Some heavy metal contents in surface water and sediment as a pollution index of El-Manzala Lake, Egypt. Journal of Basic and Environmental Sciences 4, 210–225 (2017).
30. Ismail, A. & Hettiarachchi, H. Environmental damage caused by wastewater discharge into Lake Manzala in Egypt. American Journal of Bioscience and Bioengineering 5, 141–150 (2017).
31. Beheary, M.S., Saleh, E.M. & Serag, M. Water Quality and Monitoring of Some Pollution Indicators in Lake Manzala, Egypt, Advances in Environmental Biology, 13(6), pp. 7–12 (2019).
32. Orabi, A.H., Dahab, K.A., Abdella, H.F. & El-Sehamy, M.M. Chemical-physical and ecological characterisation of a polluted coastal area: the Manzala Lake of Egypt, case study, Egyptian Journal of Geology, 63, pp. 391–406 (2019).
33. Mahmoud, A., Flefil, N., El-Sayed, S., Tahoun, U. & Goher, M. Phytoplankton and bacterial dynamics related to the physicochemical characteristics of Manzala Lake Water, Egypt. Egyptian Journal of Botany 62, 879–899 (2022).
34. Bahnasawy, M., Khidr, A.A. & Dheina, N. (2011) Assessment of heavy metal concentrations in water, plankton, and fish of Lake Manzala, Egypt, Turkish Journal of Zoology, 35(2), pp. 271–280 (2011).
35. Saeed, S. M. & Shaker, I. M. Assessment of heavy metals pollution in water and sediments and their effect on Oreochromis niloticus in the northern Delta Lakes, Egypt. in From the pharaohs to the future: Proceedings of the Eighth International Symposium on Tilapia in Aquaculture (eds. Elghobashy, H., Fitzsimmons, K. & Diab, A. S.) 475–490 (Cairo, Egypt, 2008).
36. El-Saharty, A. A. Follow up the Chemical Status of Lake Manzala water, Egypt. Egypt J Aquat Biol Fish 18, 33–46 (2014).
37. Al-Falal, A. N. A., Fadaly, E., Imam, S. & Gad, M. Monitoring and assessment of surface water quality using physicochemical parameters and indexical approaches in El Manzala Lake, Egypt. International Journal of Environmental Studies and Researches (2024).
